# Ethnobotanical Survey on Skin Whitening Prescriptions of Traditional Chinese Medicine in Taiwan

**DOI:** 10.3389/fphar.2021.736370

**Published:** 2021-11-30

**Authors:** Chien-Yu Ko, Jung Chao, Pei-Yu Chen, Shan-Yu Su, Tomoji Maeda, Chin-Yu Lin, Hung-Che Chiang, Shyh-Shyun Huang

**Affiliations:** ^1^ School of Pharmacy, China Medical University, Taichung, Taiwan; ^2^ Chinese Medicine Research Center, Department of Chinese Pharmaceutical Sciences and Chinese Medicine Resources, Master Program for Food and Drug Safety, China Medical University, Taichung, Taiwan; ^3^ Department of Cosmeceutics, China Medical University, Taichung, Taiwan; ^4^ Department of Chinese Medicine, China Medical University Hospital, Taichung, Taiwan; ^5^ School of Post-Baccalaureate Chinese Medicine, College of Chinese Medicine, China Medical University, Taichung, Taiwan; ^6^ Department of Pharmaceutical Sciences, Nihon Pharmaceutical University, Saitama, Japan; ^7^ Tsuzuki Institute for Traditional Medicine, China Medical University, Taichung, Taiwan; ^8^ Institute of New Drug Development, China Medical University, Taichung, Taiwan; ^9^ Department of Food Nutrition and Health Biotechnology, Asia University, Taichung, Taiwan

**Keywords:** skin whitening, ethnobotanical, Taiwan, traditional Chinese medicine pharmacy, traditional Taiwanese medicine

## Abstract

The increasing interest and demand for skin whitening products globally, particularly in Asia, have necessitated rapid advances in research on skin whitening products used in traditional Chinese medicine (TCM). Herein, we investigated 74 skin whitening prescriptions sold in TCM pharmacies in Taiwan. Commonly used medicinal materials were defined as those with a relative frequency of citation (RFC) > 0.2 and their characteristics were evaluated. Correlation analysis of commonly used medicinal materials was carried out to identify the core component of the medicinal materials. Of the purchased 74 skin whitening prescriptions, 36 were oral prescriptions, 37 were external prescriptions, and one prescription could be used as an oral or external prescription. After analysis, 90 traditional Chinese medicinal materials were obtained. The Apiaceae (10%; 13%) and Leguminosae (9%; 11%) were the main sources of oral and external medicinal materials, respectively. Oral skin whitening prescriptions were found to be mostly warm (46%) and sweet (53%), while external skin whitening prescriptions included cold (43%) and bitter (29%) medicinal materials. Additionally, mainly tonifying and replenishing effects of the materials were noted. Pharmacological analysis indicated that these medicinal materials may promote wound healing, treat inflammatory skin diseases, or anti-hyperpigmentation. According to the Spearman correlation analysis on interactions among medicinal materials with an RFC > 0.2 in the oral skin whitening prescriptions, *Paeonia lactiflora* Pall. (white) and *Atractylodes macrocephala* Koidz. showed the highest correlation (confidence score = 0.93), followed by *Ziziphus jujuba* Mill. (red) and *Astragalus propinquus* Schischkin (confidence score = 0.91). Seven medicinal materials in external skin whitening prescriptions with an RFC > 0.2, were classified as Taiwan *qī bái sàn* (an herbal preparation), including *Angelica dahurica* (Hoffm.) Benth. & Hook. f. ex Franch. & Sav., *Wolfiporia extensa* (Peck) Ginns, *Bletilla striata* (Thunb.) Rchb. f., *Atractylodes macrocephala* Koidz., *Ampelopsis japonica* (Thunb.) Makino, *Paeonia lactiflora* Pall. (white), and *Bombyx mori* Linnaeus. Skin whitening prescriptions included multiple traditional Chinese medicinal materials. Despite the long history of use, there is a lack of studies concerning skin whitening products, possibly due to the complex composition of traditional Chinese medicine. Further studies are required to assess the efficacy and safety of these traditional Chinese medicinal materials for inclusion in effective, safe, and functional pharmacological products.

## 1 Introduction

The global cosmetics market is undergoing an unprecedented boom due to economic development and growing aesthetic needs. Rapidly expanding Asian cosmetic markets, of which China, Japan, South Korea, and India are major consumer countries, are following in the footsteps of European and American countries with the ubiquitous use of cosmetic products, particularly skin whitening products. Moreover, a recent survey noted the increasing prevalence of males using skin whitening products in addition to females (Pillaiyar et al., 2017; Hu et al., 2020).

Skin whitening prescriptions can not only be used to lighten skin tone, but also clinically treat hyperpigmentary disorders by decreasing melanin synthesis (Gillbro and Olsson, 2011). Melanin, one of the important pigments, is synthesized in melanocytes in the basal layer of epidermis and can protect the skin from ultraviolet-induced damage (Brenner and Hearing, 2008). Epidermal melanin content is intimately associated with anthropological origins. As the level of ultraviolet radiation is higher in low-latitude regions, people in these regions have higher melanin content; conversely, melanin content is lower in people at high-latitude regions, hence they have whiter skin (Slominski et al., 2004). Melanogenesis involves the conversion of L-tyrosine to L-dihydroxyphenylalanine (L-DOPA) by tyrosinase before further conversion to L-dopaquinone. Finally, L-dopaquinone undergoes a series of chemical reactions to form melanin (Supplementary Figure S1). Tyrosinase is a rate-limiting enzyme in melanogenesis and is considered an important target for the development of therapies in treating hyperpigmentation (Sonthalia et al., 2016; Ullah et al., 2019; Zaidi et al., 2019).

At present, there are a variety of skin whitening products on the market classified as either “inhibiting melanogenesis” or “inhibiting melanogenesis and promoting melanin removal.” In Taiwan, only 13 skin whitening components (Supplementary Table S1) (Food and Drug Administration, 2014) have been approved for use in cosmetic preparations. While ascorbic acid (vitamin C) is a common component, it is unstable and easily oxidized which limits its direct use. In order to prevent premature degradation, derivatives such as magnesium ascorbyl phosphate, ascorbyl glucoside, and ascorbyl tetraisopalmitate (Balaguer et al., 2008) are often utilized. Caution must be exercised when using various skin whitening components as improper use may lead to dermatitis, erythema, burns, and other skin injuries (Nordin et al., 2021). Thus, manufacturers have begun to seek natural alternatives to develop gentle, hypoallergenic skin whitening products derived from traditional Chinese medicine (TCM).

In traditional Chinese medicine books, many words describe dark skin or spots on the face, such as miàn gǎn zèng. In addition, there are ancient descriptions regarding the use of TCM for skin whitening. The Shennong Materia Medica, an extant medicinal text published in 100 B.C., recorded that *Angelica dahurica* (Hoffm.) Benth. & Hook. f. ex Franch. & Sav. promotes skin growth and has moisturizing effects. Of additional interest is qī bái sàn, a well-known skin whitening herbal prescription. However, its composition varies among different historical dynasties, geographical regions, and environments. For example, different compositions of qī bái sàn can be found in related prescriptions such as yǒng lèi qián fāng, pǔ jì fāng, and tài píng shèng huì fāng (Table 1). To date, there is still no comprehensive study on skin whitening prescriptions in traditional Chinese medicine pharmacies in Taiwan. Therefore, the aim of this study was to examine the composition of skin whitening prescriptions sold in traditional Chinese medicine pharmacies in Taiwan to understand the usage, methods of preparation, and principles of skin whitening prescriptions in Taiwan.

**TABLE 1:** Qī bái sàn-related prescriptions in ancient books.

Name of prescription	Composition	Source
qī bái sàn (七白散)	*Atractylodes macrocephala* Koidz., *Ampelopsis japonica* (Thunb.) Makino, *Angelica dahurica* (Hoffm.) Benth. & Hook.f. ex Franch. & Sav., *Bombyx mori* Linnaeus, *Paeonia lactiflora* Pall., *Ipomoea nil* (L.) Roth, *Sauromatum giganteum* (Engl.) Cusimano & Hett	yǒng lèi qián fang (永類鈐方)
qī bái wán (七白丸)	*Atractylodes macrocephala* Koidz., *Ampelopsis japonica* (Thunb.) Makino, *Angelica dahurica* (Hoffm.) Benth. & Hook.f. ex Franch. & Sav., *Bletilla striata* (Thunb.) Rchb.f., *Bombyx mori* Linnaeus, *Wolfiporia extensa* (Peck) Ginns, *Sauromatum giganteum* (Engl.) Cusimano & Hett	pǔ jì fang (普濟方)
qī bái sàn xǐ miàn yào (七白散洗面藥)	*Atractylodes macrocephala* Koidz., *Ampelopsis japonica* (Thunb.) Makino, *Angelica dahurica* (Hoffm.) Benth. & Hook.f. ex Franch. & Sav., *Bombyx mori* Linnaeus, *Paeonia lactiflora* Pall., *Ipomoea nil* (L.) Roth, *Wolfiporia extensa* (Peck) Ginns	pǔ jì fang (普濟方)
qī bái tǐng zǐ gāo (七白挺子膏)	*Atractylodes macrocephala* Koidz., *Ampelopsis japonica* (Thunb.) Makino, *Angelica dahurica* (Hoffm.) Benth. & Hook.f. ex Franch. & Sav., *Asarum heterotropoides* F.Schmidt f. *mandshuricum* (Maxim.) Kitag., *Bletilla striata* (Thunb.) Rchb.f., *Wolfiporia extensa* (Peck) Ginns, *Sauromatum giganteum* (Engl.) Cusimano & Hett	tài píng shèng huì fang (太平聖惠方)
	(This prescription must be mixed with egg white for use)	

## 2 Materials and Methods

### 2.1 Ethical Review

The research for this study was conducted from March 2020 to April 2021. The study was approved by the Central Regional Research Ethics Committee of China Medical University (CRREC-109-125) (Supplementary Figure S2).

### 2.2 Research Process

**FIGURE 1:**
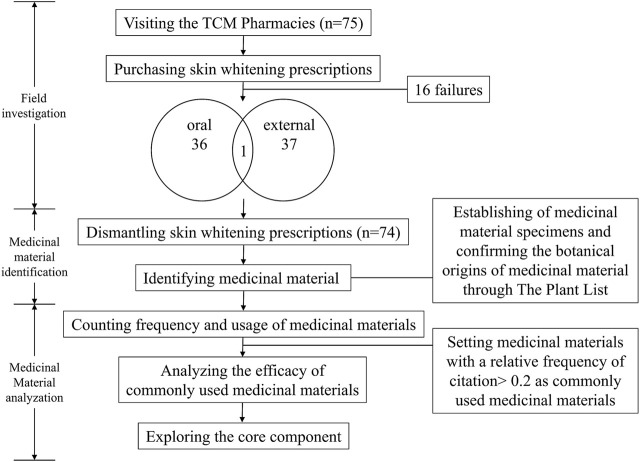
Study flowchart.

This study complied with the ethnobotanical research guidelines (Rivera et al., 2014; Mullane et al., 2015; Heinrich et al., 2018; Heinrich et al., 2020), and could mainly divided into field investigation, medicinal material identification, and analysis. The complete study methods are shown in the study flowchart (Figure 1).

#### 2.2.1 Field Investigation

**FIGURE 2:**
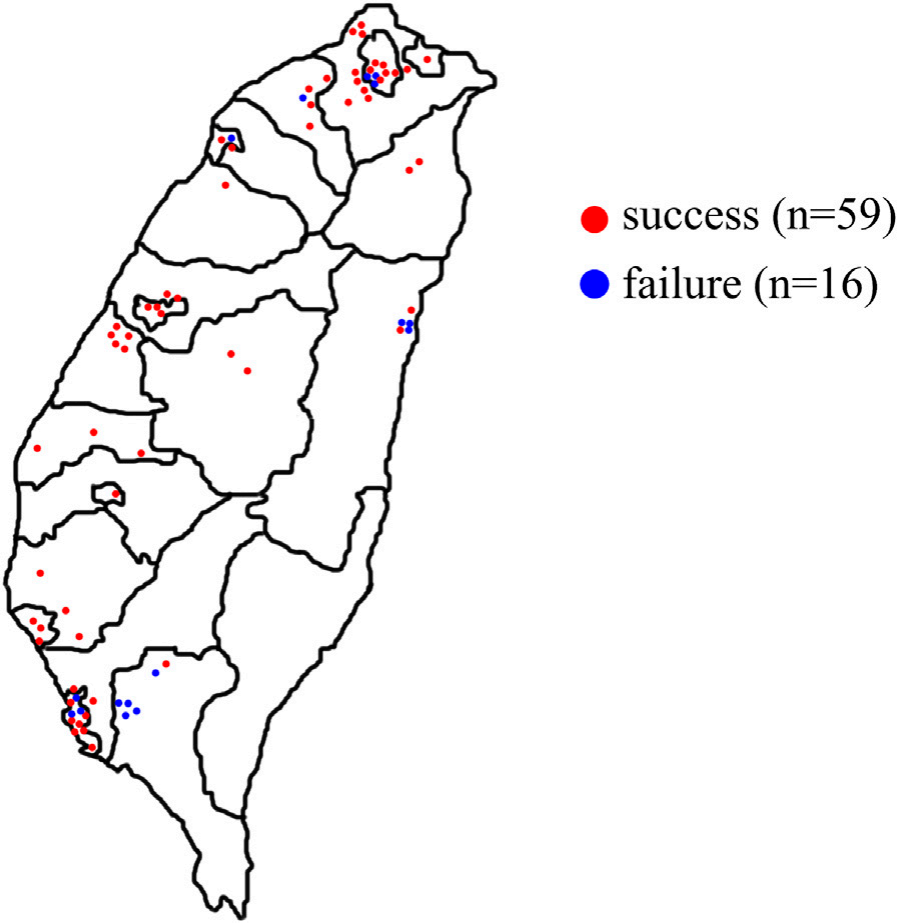
Geographical distribution of pharmacies visited for skin whitening prescriptions in Taiwan. All dots were based on latitude and longitude coordinates. Red dots indicated successful pharmacies; blue dots indicated failed pharmacies.

Taiwan is an island located at the intersection between Northeast Asia and Southeast Asia and a total area of 36,000 km^2^ (Tourism Bureau, 2021). The study was conducted over 12 months and we randomly visited the TCM pharmacies that appropriately represented the use of TCM medicine in Taiwan. The number of TCM pharmacies selected was directly proportional to the number of TCM pharmacies in each county and city published by the government (Ministry of Health and Welfare, 2015). A total of 75 TCM pharmacies were visited, included 16 pharmacies were visited but no prescription was obtained. Overall, 74 skin whitening prescriptions were obtained (with 13 TCM pharmacies providing more than one prescription) (Figure 2), including 36 oral prescriptions, 37 external prescriptions, and one prescription which could be used as an oral or external prescription.

#### 2.2.2 Identification of Medicinal Materials

**FIGURE 3:**
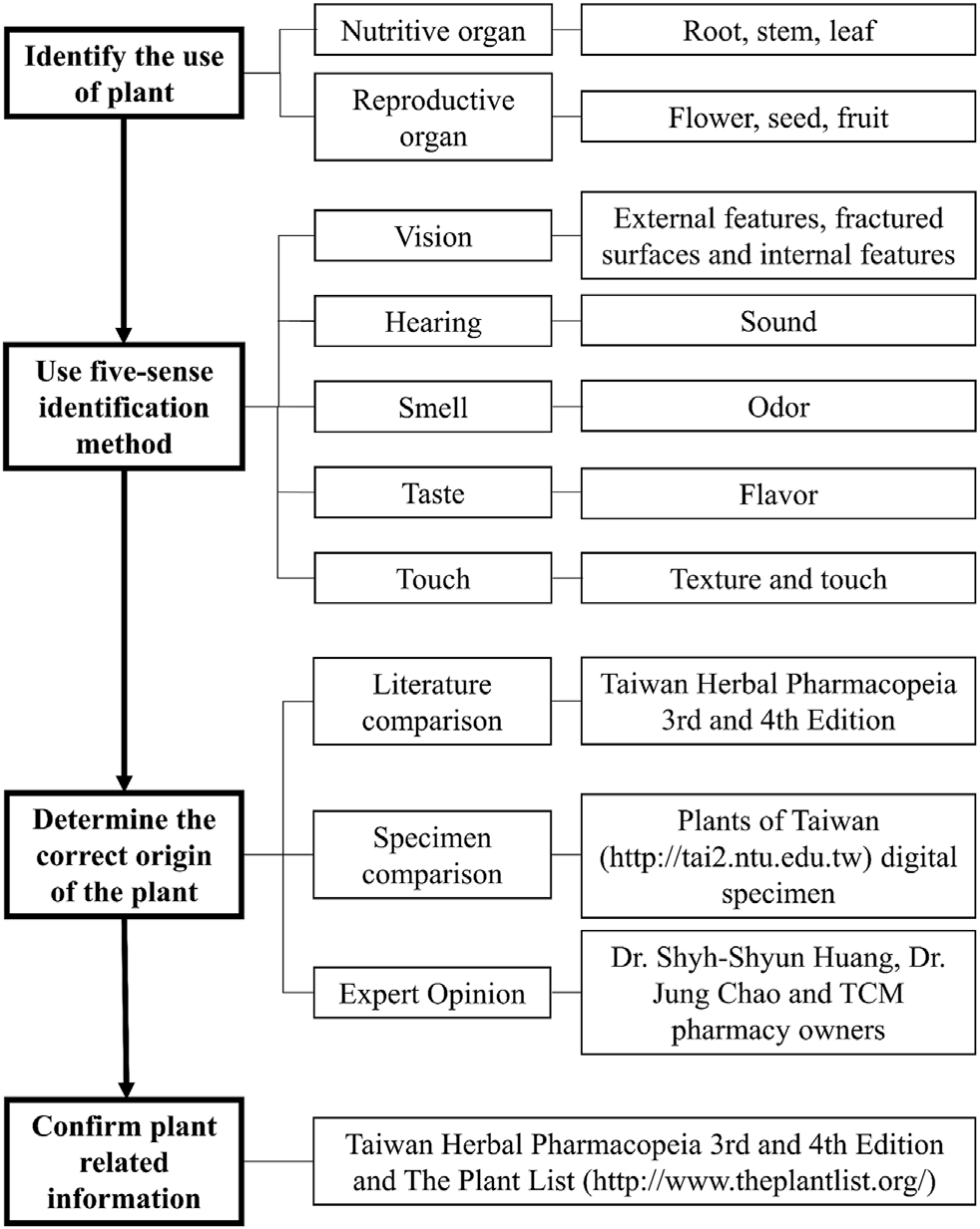
Flowchart for medicinal materials identification.

This study examined the purchased medicinal materials and performed the five-sense identification to identify the origins and plant parts of the materials, and compared them with the medicinal material standards to distinguish authentic or misused medicinal materials (Figure 3). We also photographed the materials and recorded the weight of each material. Finally, the materials were numbered and stored in the herbarium of the China Medical University, Taiwan.

#### 2.2.3 Analysis of Medicinal Materials

The various medicinal materials were analyzed and collated based on biological taxonomy, relative frequency of citation (RFC), efficacy of traditional use, and skin-related pharmacological effects. Biological taxonomy included the Scientific name of the crude drug, family, and color. The Plant List (http://www.theplantlist.org/) was used as a source of botanical information. Medicinal materials with RFC > 0.2 were defined as commonly used medicinal materials. The RFC formula was defined as follows (Vitalini et al., 2013; Dixit and Tiwari, 2020; Abbas et al., 2021):

$$\mathrm{RF}C_{i}=FC_{i}/N\left(0\underline{\leq }{\mathrm{RFC}}_{i}\underline{\leq }1\right)$$

Where $RFC_{i}$ is the relative frequency count of *i* species and it is commonly used in ethnopharmacology papers. $FC_{i}$ defines the count of prescriptions which used species *i*. N denotes the total number of prescriptions.

The medicinal materials were indexed against the Taiwan Herbal Pharmacopeia (3rd, 4th edition) (Taiwan Herbal Pharmacopeia 3rd Edition Committee, 2018; Taiwan Herbal Pharmacopeia 4th Edition Committee, 2021), Pharmacopoeia of the People’s Republic of China (Chinese Pharmacopoeia Commission, 2020), and Chinese Materia Medica (State Administration of Traditional Chinese Medicine, 1999). The effects, properties, and flavors of traditional Chinese medicine were cited from the Taiwan Herbal Pharmacopeia (3rd, 4th edition). The PubMed database was systematically searched from Jan 2010 to May 2021 for skin-related pharmacological effects, utilizing keywords such as “skin” and the scientific names of medicinal materials.

#### 2.2.4 Analysis

GraphPad Prism 9.0 (GraphPad Prism version 9.0 for Windows, GraphPad Software, San Diego, California, USA) was used to plot a heat map for Spearman correlation analysis of commonly used medicinal materials used in oral skin whitening prescriptions. The colors of the squares in the heat map were based on the visualization of Spearman correlation matrix of the two medicinal materials. The more intense red hue, the higher the correlation between the two medicinal materials. Conversely, the lighter the color, the lower the correlation between the two medicinal materials (Vacanti, 2019).

## 3 Results

### 3.1 Biological Taxonomic Characteristics of Medicinal Materials Used in Skin Whitening Prescriptions

**FIGURE 4:**
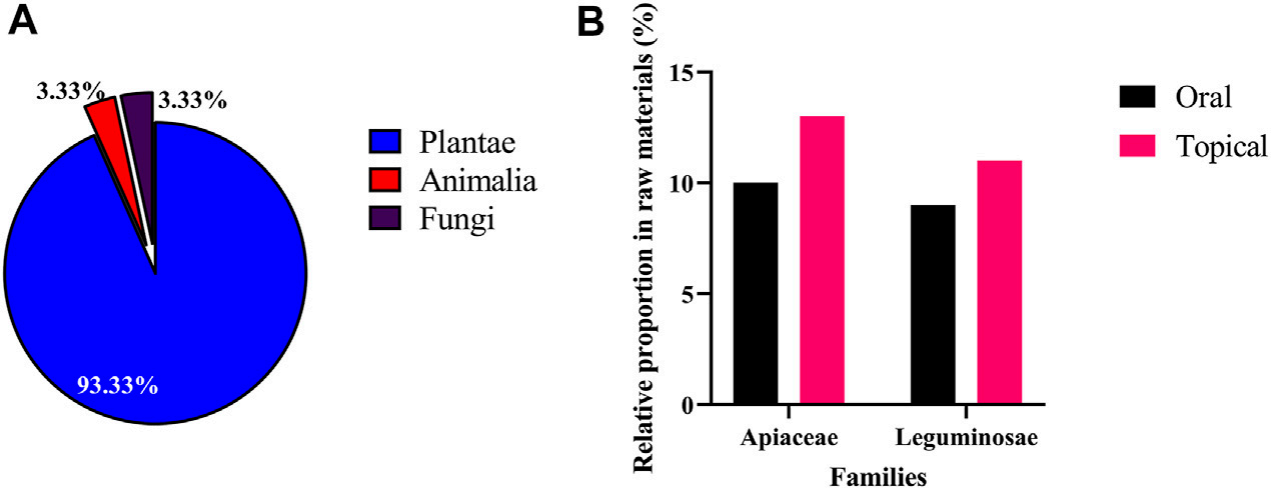
Taxonomy of 90 medicinal materials in 74 skin whitening prescriptions. **(A)** Kingdoms and **(B)** families.

During this study, 74 skin whitening prescriptions were purchased from 59 TCM pharmacies in Taiwan, of which 36 were oral prescriptions, 37 were external prescriptions, and one prescription could be used orally or externally. Oral prescription use method is to add appropriate amount of water to decoct; external prescription use method is to mash the medicinal materials, then add water, honey or milk, and apply to the face. Among the oral and external skin whitening prescriptions, 79 and 56 medicinal materials were found respectively. Overall, a total of 90 medicinal materials were obtained from the 74 prescriptions, and 6 misused medicinal materials were found (Supplementary Table S2). The majority of these medicinal materials were Plantae (93.33%), 3 medicinal materials (3.33%) were obtained from Animalia [*Bombyx mori* Linnaeus, *Crassostrea gigas* (Thunberg), and *Pteria martensii* (Dunker)] and 3 (3.33%) were from Fungi [*Tremella fuciformis*, *Wolfiporia extensa* (Peck) Ginns cum pini radix, and *Wolfiporia extensa* (Peck) Ginns] (Figure 4A).

The most used of medicinal material in oral skin whitening prescriptions was *Wolfiporia extensa* (Peck) Ginns (RFC = 0.51), followed by *Glycyrrhiza uralensis* Fisch. and *Paeonia lactiflora* Pall. (white) (RFC = 0.41), while *Angelica dahurica* (Hoffm.) Benth. & Hook. f. ex Franch. & Sav. (RFC = 0.89) was the most commonly used of medicinal material in external skin whitening prescriptions. When classified by family, the most common families in both oral and external skin whitening prescriptions were Apiaceae (10 and 13% respectively) and Leguminosae (9 and 11% respectively) (Figure 4B).

### 3.2 Analysis of Traditional Efficacy, Skin-Related Pharmacological Effects and Dosage of Commonly Used Medicinal Materials in Skin Whitening Prescriptions

Commonly used medicinal materials were defined as those with RFC > 0.2. Thirteen and seven commonly used medicinal materials were obtained from oral and external skin whitening prescriptions, respectively (Table 2).

**TABLE 2:** Medicinal properties and skin-related modern research of commonly used medicinal materials used in skin whitening prescriptions (RFC>0.2).

Scientific name/Latin name of crude drug/Local name/sample number	RFC^a^	Family	Color	Flavor and property	Traditional usage	Literature on skin modern research (PubMed)^b^
Oral						
*Wolfiporia extensa* (Peck) Ginns/Poria/白茯苓/CMU2021SWP	0.51	Polyporaceae	white	Sweet and plain; plain	Dampness-draining diuretic	Hyperpigmentation (Ho et al., 2021); inhibition of melanogenesis (Lee and Cha, 2018; Kang et al., 2019); moisturizing and increased skin barrier function (Choi et al., 2019); oxidative stress associated skin aging effects and inflammatory skin diseases (Kang et al., 2019; Fang et al., 2021)
*Glycyrrhiza uralensis* Fisch./Glycyrrhizae radix et rhizoma/甘草/CMU2021SWGrr	0.41	Leguminosae	brown	Sweet; plain	Tonifying and replenishing	Accelerate wound healing and promote neovascularization (Hao et al., 2020) anti-photoaging effects (Kim et al., 2017; Xuan et al., 2017); human dermal fibroblasts (Kim et al., 2017); inflammatory skin diseases (Jeong et al., 2015; Cha et al., 2016; Lee et al., 2020); inhibition of melanogenesis (Lim et al., 2018); protection of skin barrier (Cha et al., 2017); viral skin diseases (Wang et al., 2013a)
*Paeonia lactiflora* Pall. (white)/Paeoniae radix alba/白芍/CMU2021SWPra	0.41	Paeoniaceae	white	Bitter and sour; cold	Tonifying and replenishing	Ameliorated vascular damage (Chen et al., 2013); anti-photoaging effects (Lu et al., 2020); hyperpigmentation (Qiu et al., 2016; Ho et al., 2021); inflammatory skin diseases (Chen et al., 2011; Jeong et al., 2015; Kim et al., 2021); inhibition of melanogenesis (You et al., 2017); psoriasis (Sun et al., 2015a; Choi et al., 2015; Li et al., 2019); skin itching Zhu et al., 2019)
*Angelica dahurica* (Hoffm.) Benth. & Hook.f. ex Franch. & Sav./Angelicae dahuricae radix/白芷/CMU2021SWAdr	0.35	Apiaceae	white	Pungent; warm	Exterior-releasing	Accelerate wound healing and promote neovascularization (Bai et al., 2012; Yang et al., 2017; Yang et al., 2020a); acne (Hwang et al., 2016); inflammatory skin diseases (Lee et al., 2012; Ku et al., 2017); inhibition of melanogenesis (Kim et al., 2016); melanoma (Hwangbo et al., 2020); skin itching (Zhu et al., 2019)
*Astragalus propinquus* Schischkin/Astragali radix/黃耆/CMU2021SWApr	0.32	Leguminosae	brown	Sweet; warm	Tonifying and replenishing	Accelerate wound healing (Luo et al., 2016; Zhao et al., 2017); anti-photoaging effects (Hong et al., 2013; Berezutsky et al., 2019; Shan et al., 2019); hyperpigmentation (Tsao et al., 2017); inflammatory skin diseases (Kim et al., 2013; Deng et al., 2019)
*Angelica sinensis* (Oliv.) Diels/Angelicae sinensis radix/當歸/CMU2021SWAsr	0.32	Apiaceae	brown	Sweet and pungent; warm	Tonifying and replenishing	Accelerate wound healing (Hsiao et al., 2012; Zhao et al., 2012; Wang et al., 2013b); inflammatory skin diseases (Gong et al., 2015; Choi et al., 2016; Lee et al., 2016; Saba et al., 2016; Nam et al., 2021); skin itching (Lee at al., 2016; Zhu et al., 2019); melanoma (Gao et al., 2018)
*Coix lacryma-jobi var. ma-yuen* (Rom.Caill.) Stapf/Coicis semen/白薏仁/CMU2021SWCs	0.32	Poaceae	white	Sweet and plain; cool	Dampness-draining diuretic	Accelerate wound healing (Kalekhan et al., 2021); anti-photoaging effects (Shan et al., 2012); chapped skin and warts (Chung et al., 2011; Byun et al., 2016; Son et al., 2019); inhibition of melanogenesis (Huang et al., 2014; Amen et al., 2017)
*Ziziphus jujuba* Mill. (red)/Jujubae fructus (red)/紅棗/CMU2021SWJf	0.3	Rhamnaceae	red	Sweet; warm	Tonifying and replenishing	Accelerate wound healing (Fazio et al., 2020); anti-wrinkle (Son and Lee, 2020); melanoma (Hung et al., 2012)
*Atractylodes macrocephala* Koidz./Atractylodis macrocephalae rhizoma/白朮/CMU2021SWAmr	0.3	Compositae	white	Bitter and sweet; warm	Tonifying and replenishing	Hyperpigmentation (Ho et al., 2021); skin itching (Zhu et al., 2019)
*Dioscorea polystachya* Turcz./Dioscoreae rhizoma/白山藥/CMU2021SWDr	0.24	Dioscoreaceae	white	Sweet; plain	Tonifying and replenishing	Inflammatory skin diseases (Jegal et al., 2017; Jegal et al., 2018); skin cancer (Tsukayama et al., 2018)
*Lycium chinense* Mill./Lycii fructus/枸杞子/CMU2021SWLf	0.24	Solanaceae	red	Sweet; plain	Tonifying and replenishing	Anti-photoaging effects (Yi et al., 2013; Im et al., 2016; Li at el., 2017; Liang et al., 2018; Neves et al., 2020; Neves et al., 2021); inflammatory skin diseases (Wu et al., 2020); melanoma (Cenariu et al., 2021); moisturizing (Meng et al., 2020)
*Ophiopogon japonicus* (Thunb.) Ker Gawl./Ophiopogonis radix/麥門冬/CMU2021SWOr	0.24	Asparagaceae	white	Sweet and bitter; cold	Tonifying and replenishing	Inflammatory skin diseases (Kitahiro et al., 2018; Mainzer et al., 2019; An et al., 2020)
*Ligusticum striatum* DC./Chuanxiong rhizoma/川芎/CMU2021SWCr	0.22	Apiaceae	brown	Pungent; warm	Tonifying and replenishing	Inflammatory skin diseases (Lee et al., 2016; Yan et al., 2020); skin itching (Zhu et al., 2019)
External						
*Angelica dahurica* (Hoffm.) Benth. & Hook.f. ex Franch. & Sav./Angelicae dahuricae radix/白芷/CMU2021SWAdr	0.89	Apiaceae	white	Pungent; warm	Exterior-releasing	Accelerate wound healing and promote neovascularization (Bai et al., 2012; Yang et al., 2017; Yang et al., 2020b): acne (Hwang et al., 2016); inflammatory skin diseases (Lee et al., 2012; Ku et al., 2017); inhibition of melanogenesis (Kim et al., 2016); melanoma (Hwangbo et al., 2020); skin itching (Zhu et al., 2019)
*Wolfiporia extensa* (Peck) Ginns/Poria/白茯苓/CMU2021SWP	0.82	Polyporaceae	white	Sweet and plain; plain	Dampness-draining diuretic	Hyperpigmentation (Ho et al., 2021); inhibition of melanogenesis (Lee and cha, 2018; Kang et al., 2019); moisturizing and increased skin barrier function (Choi et al., 2019); oxidative stress associated skin aging effects (Kang et al., 2019; Fang et al., 2021)
*Bletilla striata* (Thunb.) Rchb.f./Bletillae rhizoma/白及/CMU2021SWBr	0.58	Orchidaceae	white	Bitter, sweet, and astringent; cold	Hemostatic	Accelerate wound healing (Yu et al., 2011; He et al., 2017; Song et al., 2017; Zhang et al., 2019; Yang et al., 2020a); chapped skin (He et al., 2017); hyperpigmentation (Ho et al., 2021)
*Atractylodes macrocephala* Koidz./Atractylodis macrocephalae rhizoma/白朮/CMU2021SWAmr	0.53	Compositae	white	Bitter and sweet; warm	Tonifying and replenishing	Hyperpigmentation (Ho et al., 2021); skin itching (Zhu et al., 2019)
*Ampelopsis japonica* (Thunb.) Makino/Ampelopsis radix/白蘞/CMU2021SWAr	0.37	Vitaceae	white	Bitter and pungent; cold	Heat-clearing	Accelerate wound healing and promote neovascularization (Lee et al., 2015); hyperpigmentation (Fong and Tong, 2012; Ho et al., 2021)
*Paeonia lactiflora* Pall. (white)/Paeoniae radix alba/白芍/CMU2021SWPra	0.32	Paeoniaceae	white	Bitter and sour; cold	Tonifying and replenishing	Ameliorated vascular damage (Chen et al., 2013); anti-photoaging effect (Lu et al., 2020); hyperpigmentation (Qiu et al., 2016; Ho et al., 2021); inflammatory skin diseases (Chen et al., 2011; Jeong et al., 2015; Kim et al., 2020); inhibition of melanogenesis (You et al., 2017); psoriasis (Sun et al., 2015b; Choi et al., 2015; Li et al., 2019); skin itching (Zhu et al., 2019)
*Bombyx mori* Linnaeus/Bombyx batryticatus/白殭蠶/CMU2021SWBb	0.26	Bombycidae	white	Salty and pungent; plain	Liver-pacifying and wind-extinguishing	Accelerate wound healing (Tariq et al., 2021)

a RFC, relative frequency of citation.

b Literature on skin modern research (PubMed) included pharmacological effects, clinical research and intervention research.

**FIGURE 5:**
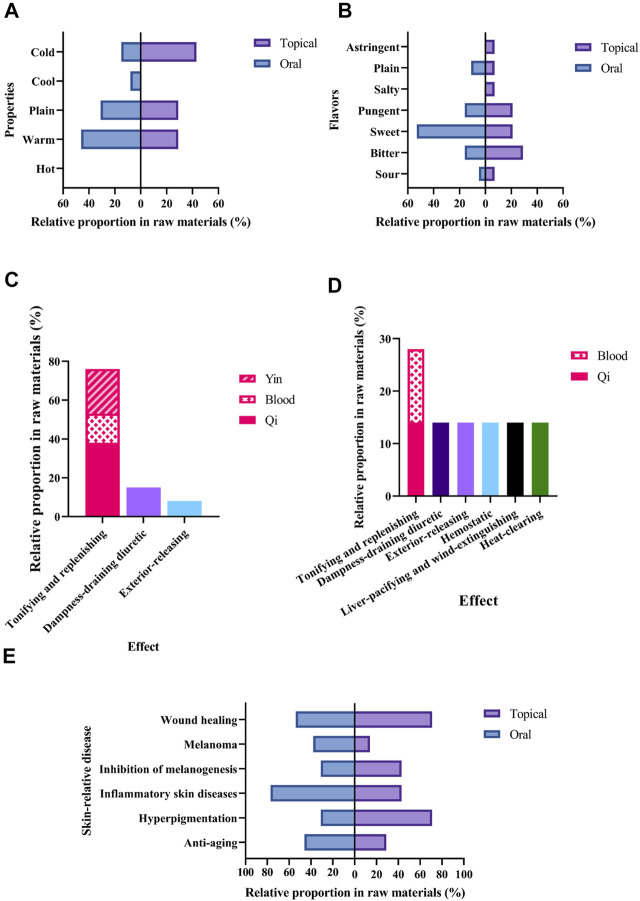
Characteristics of medicinal materials with an RFC > 0.2 in skin whitening prescriptions. **(A)** Properties and **(B)** Flavors. Histogram of traditional efficacy classifications. **(C)** oral prescriptions and **(D)** external prescriptions. **(E)** Modern research related to skin.

With regards to the properties (Figure 5A), commonly used medicinal materials used in oral skin whitening prescriptions were mostly warm (46%) and plain (31%), while those used in external were mostly cold (43%), followed by warm (29%) and plain (29%). About the flavors (Figure 5B), commonly used medicinal materials used in oral skin whitening prescriptions were mostly sweet (53%), while those used in external were mostly bitter (29%).

About classification by traditional effect (Figures 5C,D), commonly used medicinal materials used in both oral and external skin whitening prescriptions were mostly tonifying and replenishing. Integrated the modern research related to skin, including pharmacological effects, clinical studies and intervention studies, it is found that most of these medicinal materials could promote wound healing, treat inflammatory skin diseases, or anti-hyperpigmentation (Figure 5E).

**FIGURE 6:**
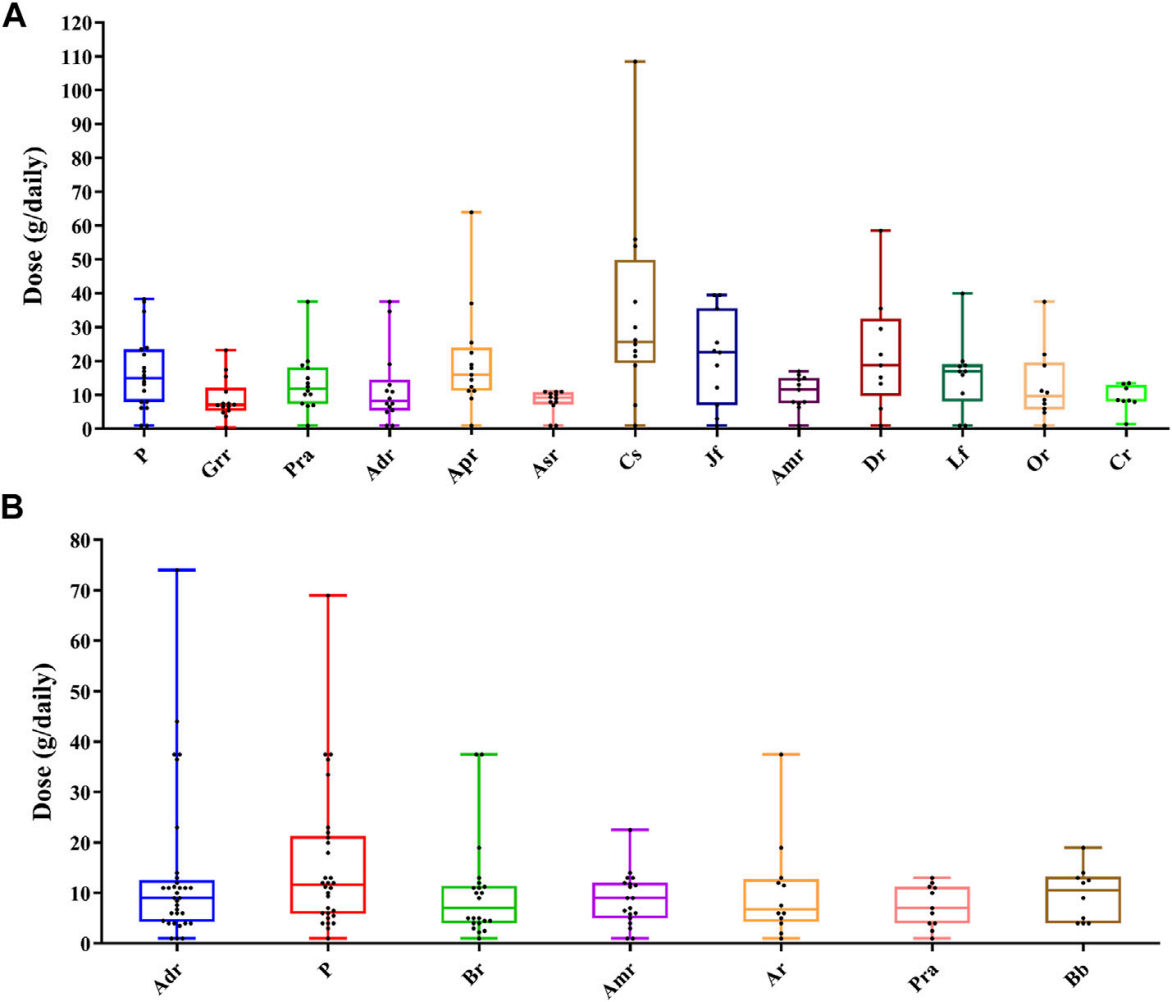
Box plots of dose ranges of commonly used medicinal materials. The top line represents the maximum value, and the bottom line represents the minimum value; the bottom of each box represents the first quartile (Q1), the middle line represents the second quartile (Q2), and the top of each box represents the third quartile (Q3). The black dots represent the doses in the collected samples. **(A)** Oral prescriptions and **(B)** external prescriptions. Adr, *Angelica dahurica* (Hoffm.) Benth. & Hook. f. ex Franch. & Sav.; Amr, *Atractylodes macrocephala* Koidz; Apr, *Astragalus propinquus* Schischkin; Ar, *Ampelopsis japonica* (Thunb.) Makino; Asr, *Angelica sinensis* (Oliv.) Diels; Cr, *Ligusticum striatum* DC.; Cs, *Coix lacryma-jobi* var. *ma-yuen* (Rom.Caill.) Stapf; Dr, *Dioscorea polystachya* Turcz.; Grr, *Glycyrrhiza uralensis* Fisch.; Jf, *Ziziphus jujuba* Mill. (red); Lf, *Lycium chinense* Mill.; Or, *Ophiopogon japonicus* (Thunb.) Ker Gawl.; P, *Wolfiporia extensa* (Peck) Ginns; Pra, *Paeonia lactiflora* Pall. (white).

The analysis of the various medicinal materials dosage used in prescriptions is presented in Supplementary Table S3. In commonly used oral medicinal materials (Figure 6A), the average dose of *Coix lacryma-jobi* var. *ma-yuen* (Rom.Caill.) Stapf was the highest and that *Glycyrrhiza uralensis* Fisch. was the lowest. *Coix lacryma-jobi* var. *ma-yuen* (Rom.Caill.) Stapf showed the largest dose difference across the various TCM pharmacies, while dose differences of the *Angelica sinensis* (Oliv.) Diels were the smallest. In commonly used external medicinal materials (Figure 6B), the average dosage of *Wolfiporia extensa* (Peck) Ginns was the highest, while the *Bletilla striata* (Thunb.) Rchb. f. and *Paeonia lactiflora* Pall. (white) were the lowest. The dosages of *Angelica dahurica* (Hoffm.) Benth. & Hook. f. ex Franch. & Sav. had the largest differences across various TCM pharmacies, while the dosages of the *Paeonia lactiflora* Pall. (white) exhibited the smallest difference.

### 3.3 Correlation Analysis of Commonly Used Medicinal Materials Used in Oral Skin Whitening Prescriptions

**FIGURE 7:**
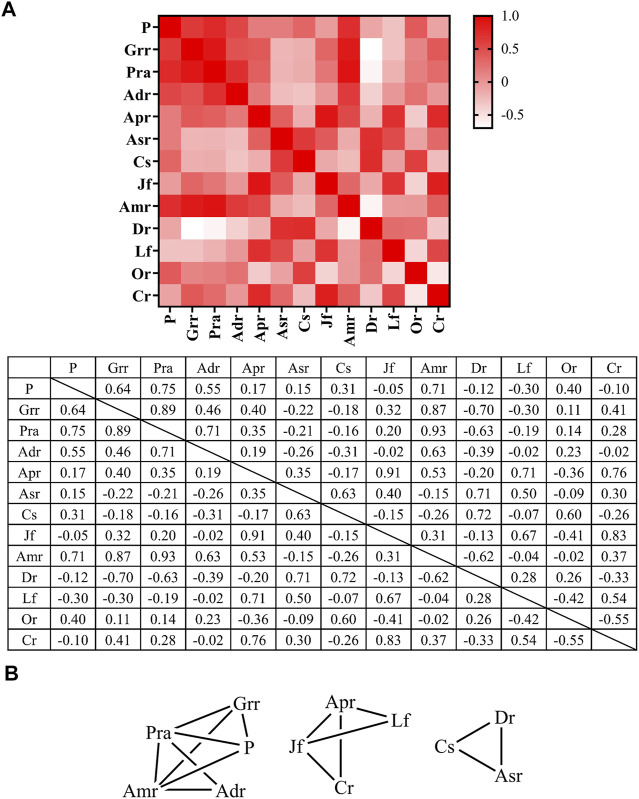
Spearman correlation analysis of commonly used medicinal materials used in oral skin whitening prescriptions. **(A)** Heat map and **(B)** Network map. Adr, *Angelica dahurica* (Hoffm.) Benth. & Hook. f. ex Franch. & Sav.; Amr, *Atractylodes macrocephala* Koidz.; Apr, *Astragalus propinquus* Schischkin; Asr, *Angelica sinensis* (Oliv.) Diels; Cr, *Ligusticum striatum* DC.; Cs, *Coix lacryma-jobi* var. *ma-yuen* (Rom.Caill.) Stapf; Dr, *Dioscorea polystachya* Turcz.; Grr, *Glycyrrhiza uralensis* Fisch.; Jf, *Ziziphus jujuba* Mill. (red); Lf, *Lycium chinense* Mill.; Or, *Ophiopogon japonicus* (Thunb.) Ker Gawl.; P, *Wolfiporia extensa* (Peck) Ginns; Pra, *Paeonia lactiflora* Pall. (white).

Spearman correlation analysis was performed for commonly used medicinal materials in oral skin whitening prescriptions and a heatmap was plotted (Figure 7A). The highest correlation was detected between *Paeonia lactiflora* Pall. (white) and *Atractylodes macrocephala* Koidz. (confidence score = 0.93), followed by the correlation between *Ziziphus jujuba* Mill. (red) and *Astragalus propinquus* Schischkin (confidence score = 0.91). In contrast, low correlation was observed between *Dioscorea polystachya* Turcz. and *Glycyrrhiza uralensis* Fisch (confidence score = −0.7). *Dioscorea polystachya* Turcz. also demonstrated a low correlation with *Paeonia lactiflora* Pall. (white) and *Atractylodes macrocephala* Koidz. Therefore, *Dioscorea polystachya* Turcz. is less likely to be present when *Glycyrrhiza uralensis* Fisch., *Paeonia lactiflora* Pall. (white), or *Atractylodes macrocephala* Koidz. are present. When network analysis was performed on medicinal materials with RFC > 0.2 in oral skin whitening prescriptions (Figure 7B) and two medicinal materials with confidence score >0.6 were connected by lines, it was found that *Paeonia lactiflora* Pall. (white) and *Atractylodes macrocephala* Koidz. frequently appeared together with *Angelica dahurica* (Hoffm.) Benth. & Hook. f. ex Franch. & Sav. or *Wolfiporia extensa* (Peck) Ginns and *Glycyrrhiza uralensis* Fisch.; *Ziziphus jujuba* Mill. (red) and *Astragalus propinquus* Schischkin were used in combination with *Lycium chinense* Mill. or *Ligusticum striatum* DC.; and *Angelica sinensis* (Oliv.) Diels, *Coix lacryma-jobi* var. *ma-yuen* (Rom.Caill.) Stapf, and *Dioscorea polystachya* Turcz. was a prescription. These combinations could be used as a reference for oral skin whitening prescriptions.

### 3.4 Venn Diagram Analysis of Commonly Used Medicinal Materials Used in External Skin Whitening Prescriptions

**FIGURE 8:**
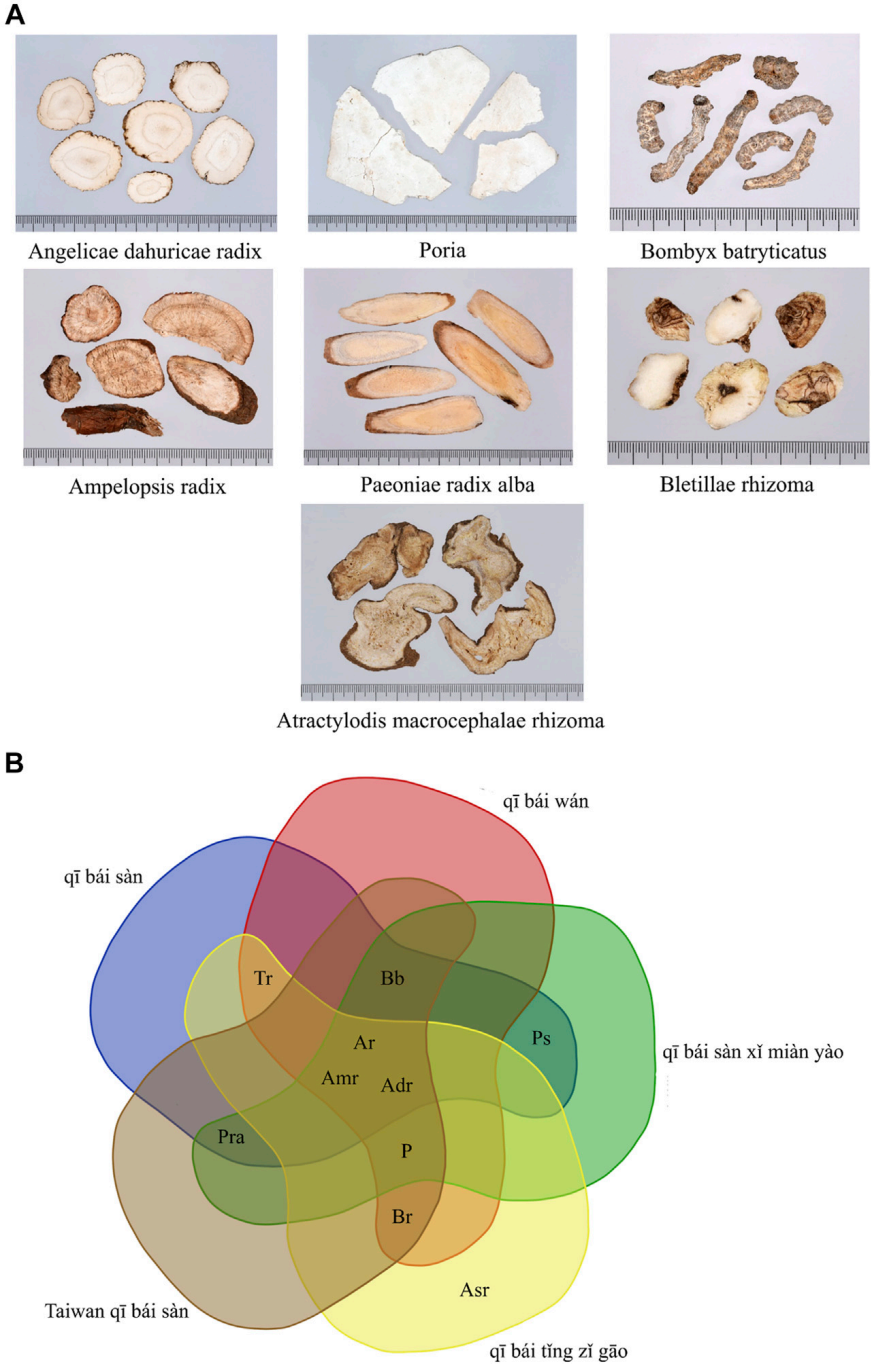
**(A)** Pictures of medicinal materials of Taiwan *qī bái sàn*. **(B)** Venn diagram of *qī bái sàn*-related prescriptions in ancient books and Taiwan *qī bái sàn*. Adr, *Angelica dahurica* (Hoffm.) Benth. & Hook. f. ex Franch. & Sav.; Amr, *Atractylodes macrocephala* Koidz.; Ar, *Ampelopsis japonica* (Thunb.) Makino; Asr, *Asarum heterotropoides* F. Schmidt f. *mandshuricum* (Maxim.) Kitag.; Bb, *Bombyx mori* Linnaeus; Br, *Bletilla striata* (Thunb.) Rchb. f.; P, *Wolfiporia extensa* (Peck) Ginns; Pra, *Paeonia lactiflora* Pall. (white); Ps, *Ipomoea nil* (L.). Roth; Tr, *Sauromatum giganteum* (Engl.) Cusimano & Hett.

In this study, medicinal materials with RFC >0.2 in external skin whitening prescriptions were defined as Taiwan *qī bái sàn* (Figure 8A). Taiwan qī bái sàn consists of *Angelica dahurica* (Hoffm.) Benth. & Hook. f. ex Franch. & Sav., *Wolfiporia extensa* (Peck) Ginns, *Bletilla striata* (Thunb.) Rchb. f., *Atractylodes macrocephala* Koidz., *Ampelopsis japonica* (Thunb.) Makino, *Paeonia lactiflora* Pall. (white), and *Bombyx mori* Linnaeus. Venn diagram analysis of these medicinal materials with qī bái sàn-related prescriptions in yǒng lèi qián fāng, pǔ jì fāng, and tài píng shèng huì fāng found that Taiwan qī bái sàn is the addition and subtraction formula from the qī bái sàn mentioned in ancient books (Figure 8B).

### 3.5 Misuse of Medicinal Materials in Skin Whitening Prescriptions

Due to the wide variety of traditional Chinese medicinal materials, some medicinal materials may have the same vernacular name but are composed of different materials, whereas some medicinal materials may have different names but same origin. During integration and analysis of medicinal materials used in skin whitening prescriptions, it was found that *Ampelopsis japonica* (Thunb.) Makino, *Astragalus propinquus* Schischkin, *Reynoutria multiflora* (Thunb.) Moldenke, *Rosa rugosa* Thunb., *Scutellaria baicalensis* Georgi, and *Tribulus terrestris* L. had misused sound alike or look alike medicinal materials (Table 3).

**TABLE 3:** Summary of medicinal materials that tend to be misused in skin whitening prescriptions.

Latin name of crude drug	Authentic or misused	Scientific name	Family	Look alike or sound alike^a^	Frequency/Use ratio (%)
Ampelopsis radix	authentic	*Ampelopsis japonica* (Thunb.) Makino	Vitaceae	look alike	11/73%
	misused	*Momordica cochinchinensis* (Lour.) Spreng	Cucurbitaceae		4/27%
Astragali radix	authentic	*Astragalus propinquus* Schischkin [*Astragalus membranaceus* (Fisch.) Bunge]^b^	Leguminosae	look alike	1/7%
	misused	*Hedysarum polybotrys* Hand. -Mazz	Leguminosae		13/93%
Reynoutriae multiflorae radix	authentic	*Reynoutria multiflora* (Thunb.) Moldenke [*Polygonum multiflorum* Thunb.]^c^	Polygonaceae	look alike	0/0%
	misused	*Pteroxygonum giraldii* Dammer and Diels	Polygonaceae		1/100%
Rosae rugosae flos	authentic	*Rosa rugosa* Thunb	Rosaceae	look alike	1/50%
	misused	*Rosa chinensis* Jacq	Rosaceae		1/50%
Scutellariae radix	authentic	*Scutellaria baicalensis* Georgi	Lamiaceae	look alike	3/60%
	misused	*Scutellaria amoena* C.H.Wright	Lamiaceae		2/40%
Tribuli fructus	authentic	*Tribulus terrestris* L.	Zygophyllaceae	sound alike	5/83%
	misused	*Astragalus complanatus* Bunge	Leguminosae		1/17%

a “Look alike” refers to the similar appearance of two confusing medicinal materials, thus causing misuse. “Sound alike” means that the local names of two confusing medicinal materials are similar in pronunciation, which causes misuse.

b *Astragalus membranaceus* (Fisch.) bunge is a commonly used synonym of *astragalus propinquus* schischkin.

c *Polygonum multiflorum* Thunb. is a commonly used synonym of *Reynoutria multiflora* (Thunb.) moldenke.

## 4 Discussion

### 4.1 Field Investigation Sites

In this study, field investigation was employed to study skin whitening prescriptions sold in TCM pharmacies in Taiwan. Field investigations are mostly used in sociology, geography, or cultural studies and was previously employed to examine the drug treatment habits for certain diseases in some regions, such as traditional Chinese medicine composition used in galactagogues prescriptions (Chao et al., 2020), herbal composition of Qīng-Căo-Chá tea (Huang et al., 2020), and medicinal materials used for hypertension (Baharvand-Ahmadi et al., 2016). Traditional Chinese Medicine, the mainstay of Asian culture, is a form of experience-based therapies, and is a medical care system for diagnosing, preventing, and treating diseases (Xu et al., 2013). Therefore, traditional Chinese pharmacies in Taiwan are important sites for retaining TCM culture.

### 4.2 Types and Biological Taxonomic Characteristics of Medicinal Materials in Skin Whitening Prescriptions

This study on the composition of skin whitening prescriptions used in Taiwan found that most medicinal materials were from Apiaceae, including *Angelica dahurica* (Hoffm.) Benth. & Hook. f. ex Franch. & Sav., *Angelica sinensis* (Oliv.) Diels and *Ligusticum striatum* DC., followed by Leguminosae, including *Glycyrrhiza uralensis* Fisch. and *Astragalus propinquus* Schischkin. Apiaceae and Leguminosae plants can inhibit tyrosinase activity, thereby reducing melanogenesis. These plants are rich in phenolic compounds and flavonoids that are proven to have significant antioxidant activity. The previous experiments also showed that they can inhibit matrix metalloproteinases (MMPs), delay skin photoaging, and stimulate keratinocyte and fibroblast migration, which has significant effects on skin regeneration (Tundis et al., 2015; Waqas et al., 2015; Zofia et al., 2020).

Melanin is an important pigment that determines skin, hair, and eye colors, and can be mainly divided into pheomelanin and eumelanin (Zanetti et al., 2001). Melanin synthesis is intimately associated with tyrosinase. Pigmentation is an important photoprotective factor, and its regulatory mechanism is extremely complex and still not completely understood. However, a large volume of data shows that ultraviolet-induced DNA damage and its repair will activate tyrosinase in melanocytes, resulting in melanogenesis (Gilchrest and Eller, 1999; Brenner and Hearing, 2008; Lai et al., 2018). Therefore, inhibition of tyrosinase can inhibit melanogenesis.

### 4.3 Analysis of Effects and Pharmacology of Commonly Used Medicinal Materials Used in Skin Whitening Prescriptions

TCM has a unique theory where medicinal materials are classified by properties (hot, warm, plain, cool, and cold) and flavors (sour, bitter, sweet, pungent, salty, plain, and astringent). Most TCM materials comprise a combination of flavors (Liao et al., 2008). The ^1^H-NMR spectrum was used to identify the properties of the medicinal materials, and it was found that their ingredients were very different (Zhang et al., 2020). A previous report showed that warm and hot medicinal materials can regulate the immune system; cold and cool medicinal materials can inhibit cell growth and proliferation (Liang et al., 2013); sweet medicinal materials have supplementation, moderation, and harmonization effects (He et al., 2012), while bitter medicinal materials mostly contain alkaloids with anti-inflammatory effects (Chen et al., 2015). The results of this study found that commonly used medicinal materials used in oral skin whitening prescriptions are mostly warm and sweet while those used in external skin whitening prescriptions are mostly cold and bitter. In combination with previous studies, it can be deduced that oral skin whitening prescriptions mostly focus on immune regulation while external skin whitening prescriptions focus on inflammation alleviation.

### 4.4 Analysis of Commonly Used Medicinal Materials Used in Oral and External Skin Whitening Prescriptions

*Wolfiporia extensa* (Peck) Ginns, *Paeonia lactiflora* Pall. (white), *Angelica dahurica* (Hoffm.) Benth. & Hook. f. ex Franch. & Sav., and *Atractylodes macrocephala* Koidz. are commonly used medicinal materials used in oral and external skin whitening prescriptions. *Wolfiporia extensa* (Peck) Ginns regulates tyrosinase activity to inhibit melanogenesis (Lee and Cha, 2018). In past studies had found that *Paeonia lactiflora* Pall. (white) can be used to treat allergic dermatitis and reduce facial wrinkles. Paeoniflorin in *Paeonia lactiflora* Pall. (white) can reduce the expression of microphthalmia-associated transcription factor (MITF) and melanogenic enzymes (including tyrosinase, TRP-1, and TRP-2) by regulating the p38 MAPK pathway, thereby inhibiting melanogenesis (Qiu et al., 2016). *Angelica dahurica* (Hoffm.) Benth. & Hook. f. ex Franch. & Sav. is a good immunomodulatory agent, and it can significantly increase phagocytosis and the secretion of cytokines by macrophages (Wang et al., 2021). It can also inhibit melanogenesis. It is involved inhibition of tyrosinase synthesis, but it does not inhibit tyrosinase activity (Cho et al., 2006). A study highlighted that the extract of *Atractylodes macrocephala* Koidz. has immune-enhancing activities (Sun et al., 2015a). Its active ingredients are Atractylenolide I and 14-acetoxy-12-senecioyloxytetradeca-2E, 8E, 10E-trien-4,6-diyn-1-ol. They are both alkaloids that demonstrate anti-inflammatory effects and are tyrosinase inhibitors (Li et al., 2007; Ye et al., 2010).

### 4.5 Misuse of Medicinal Materials in Skin Whitening Prescriptions

**FIGURE 9:**
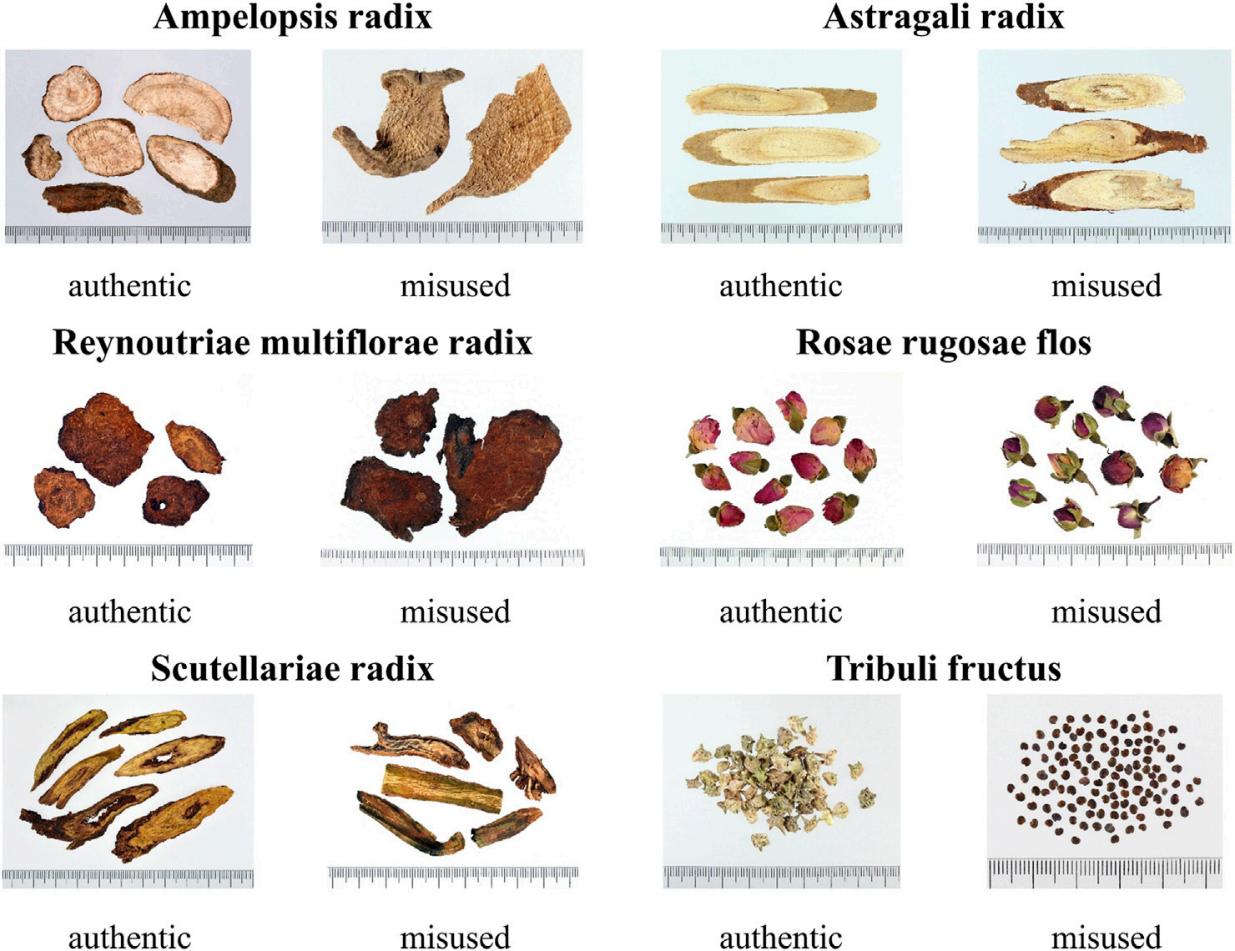
Pictures of authentic or misused medicinal materials.

The Taiwan Herbal Pharmacopeia is a codex of management regulations for TCM in Taiwan. The medicinal materials recorded in monographs should comply with the pharmacopeia standards before being manufactured, sold, and dispensed for medical treatment and health care. Medicinal materials recorded in the Taiwan Herbal Pharmacopeia are defined as authentic medicinal materials, while the origins of medicinal materials not included in the pharmacopeia are regarded as misused or fake medicinal materials (Taiwan Herbal Pharmacopeia 3rd Edition Committee, 2018; Taiwan Herbal Pharmacopeia 4th Edition Committee, 2021). Although the government releases information about authentic medicinal materials every year, medicinal materials’ misuse is still commonly seen in markets in Taiwan. Such misused materials were also found in skin whitening prescriptions that were collected in this study, including medicinal materials that look alike, which refers to the similar appearance of two confusing medicinal materials, thus causing misuse, such as *Momordica cochinchinensis* (Lour.) Spreng. that was used instead of *Ampelopsis japonica* (Thunb.) Makino, *Hedysarum polybotrys* Hand-Mazz. that was used instead of *Astragalus propinquus* Schischkin*, Scutellaria amoena* C.H.Wright that was used instead of *Scutellaria baicalensis* Georgi*, Pteroxygonum giraldii* Dammer & Diels that was used instead of *Reynoutria multiflora* (Thunb.) Moldenke, and *Rosa chinensis* Jacq. that was used instead of *Rosa rugosa* Thunb.; and medicinal materials that sound alike, which means that the local names of two confusing medicinal materials are similar in pronunciation, thus causing misuse, such as *Astragalus complanatus* Bunge that was used instead of *Tribulus terrestris* L (Figure 9). Even though misused medicinal materials in skin whitening prescriptions do not seem to cause immediate harm to the human body, there is no way to prove whether they will have adverse interactions with other medicinal materials. Therefore, simple and clear pictures showing that appearance and properties of medicinal materials should be created, and rapid, simple, and convenient identification methods should be developed to disseminate knowledge for identification of misused medicinal materials and remind traditional Chinese medicine users to be cautious and prevent misuse of medicinal materials.

### 4.6 Limitations and Future Directions

This study had certain limitations that should be addressed in future research. The first limitation is that this study was a field survey study, which only investigated skin whitening prescriptions sold in Chinese medicine stores in Taiwan. The skin whitening prescriptions investigated in this study reflect local usage in Taiwan. Further research will be needed to examine the whitening prescriptions used in traditional Chinese medicine in other regions. The second limitation is that this study only analyzed the core medicinal materials and dosages of the prescriptions but not the efficacy of these botanical drugs. Therefore, animal or cell experiments can be used in future research to verify the effectiveness of the skin whitening medicinal materials or prescriptions. The last limitation is that the Spearman correlation analysis only shows the correlations between prescription drugs and not the drug interactions between them. When discussing prescriptions in future research, we may have to consider both the theoretical significance of traditional Chinese medicine and the practical significance. We should also conduct *in vivo* and *in vitro* experiments to further explore the efficacy and the interactions between the medicinal materials.

## 5 Conclusion

In Asian countries, whiter skin color is synonymous with beauty for women and many Asian women look for natural and without side effects skin whitening products in order to reduce cutaneous pigmentation. This study is the first ethnobotanical survey on skin whitening prescriptions collected from TCM pharmacies in Taiwan. The purpose is to preserve the use of TCM for skin whitening in Taiwan. Although the use of TCM in skin whitening has been widely recorded and many skin whitening medicinal materials were collected from TCM pharmacies in this study, the ingredients of TCM are extremely complex and tends to be affected by many factors, may also be used in misused medicinal materials. Moreover, the efficacy and safety of most medicinal materials have not been scientifically validated. Further studies are needed to support the conversion of traditional skin whitening prescriptions to effective functional products. Thus, the results of this study may provide significant foundational data for subsequent studies The Royal Botanic Gardens, 2013, Li et al., 2017, Institute of Ecology et al., 2021.

## Data Availability

The original contributions presented in the study are included in the article/Supplementary Material, further inquiries can be directed to the corresponding author.
