# Supplementary material for: Ethnobotanical Survey on Skin Whitening Prescriptions of Traditional Chinese Medicine in Taiwan
Source: Front Pharmacol. 2021 Nov 30;12:736370. doi: 10.3389/fphar.2021.736370 (PMC8670535; doi:10.3389/fphar.2021.736370)
Supplement: Supplementary file 1 [file DataSheet1.docx]

Supplementary Data

| **Table S1. Skin whitening components that can be used in cosmetics in Taiwan** | |
| --- | --- |
| Component | Limit or concentration used (%) |
| Magnesium ascorbyl phosphate | 3 |
| Ascorbyl glucoside | 2 |
| Kojic acid | 2 |
| Arbutin | 7  (Product contains the impurity hydroquinone<20 ppm) |
| Sodium ascorbyl phosphate | 3 |
| Ellagic acid | 0.5 |
| Chamomile ET | 0.5 |
| Tranexamic acid | 2-3 |
| Potassium methoxysalicylate | 1-3 |
| 3-O-Ethyl ascorbic acid | 1-2 |
| 5,5’-Dipropyl-biphenyl-2,2’-diol | 0.5 |
| Cetyl tranexamate HCl | 3 |
| Ascorbyl tetraisopalmitate | 3 |

| 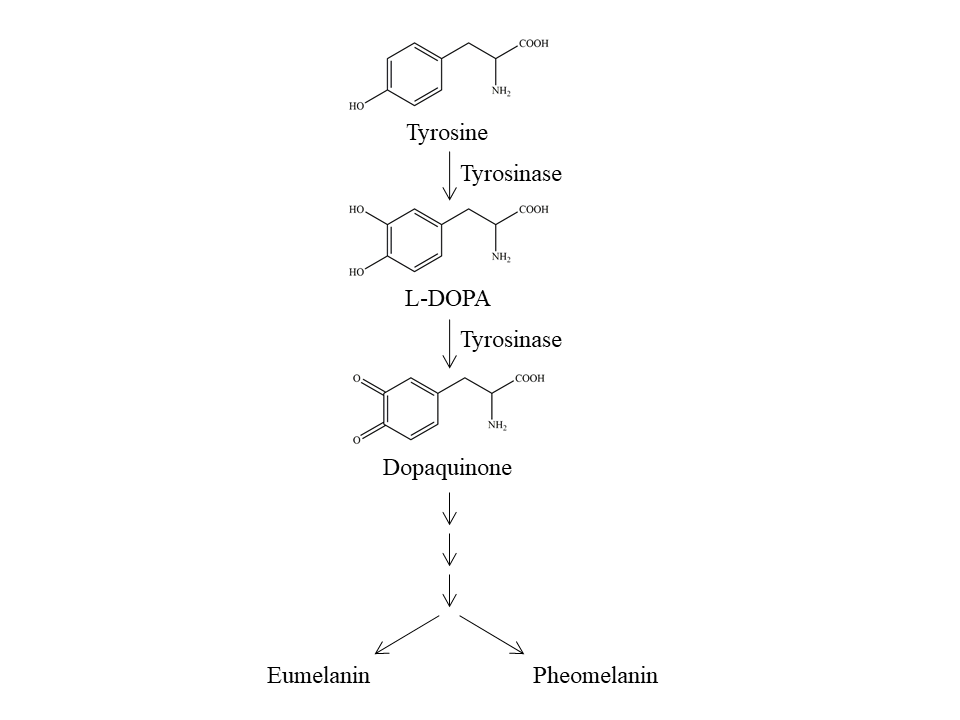 |
| --- |
| **Figure S1. Melanogenesis pathway** |

| 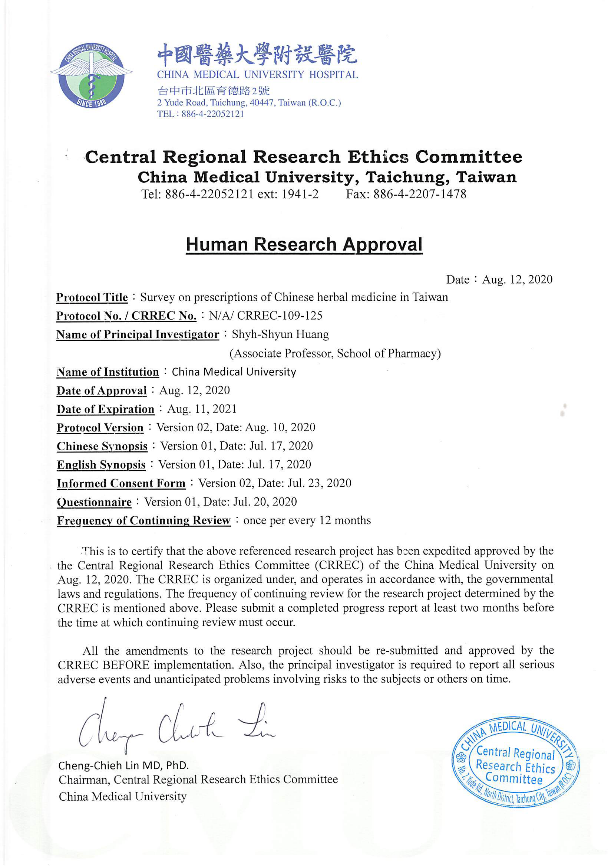 |
| --- |
| **Figure S2. Study approval letter from the Central Regional Research Ethics Committee of China Medical University Review (CRREC-109-125)** |

| **Table S2. 90 medicinal materials in 74 skin-whitening prescriptions.** | | | | | | |
| --- | --- | --- | --- | --- | --- | --- |
| Scientific name/Latin name of crude drug/Local name/sample number | Kingdom | Family | Flavor and property | Usage | Dosage (g/daily) | |
|  |  |  |  |  | Oral | External |
| *Syzygium aromaticum* (L.) Merr. & L.M.Perry/Caryophylli flos/丁香/CMU2021SWSaf | Plantae | Myrtaceae | pungent; warm | O/E | 1 | 6-12 |
| *Panax ginseng* C.A.Mey./Ginseng radix et rhizoma/人參/CMU2021SWPgrr | Plantae | Araliaceae | sweet, bitter; warm | O/E | 1.9-11.5 | 8-12 |
| *Foeniculum vulgare* Mill./Foeniculi fructus/小茴香/CMU2021SWFvf | Plantae | Apiaceae | pungent; warm | O/E | 4.5 | 4.5 |
| *Kaempferia galanga* L./Kaempferiae rhizoma/山柰/CMU2021SWKgr | Plantae | Zingiberaceae | pungent; warm | O/E | 1-7.4 | 7 |
| *Dioscorea polystachya* Turcz./Dioscoreae rhizoma/白山藥/CMU2021SWDr | Plantae | Dioscroeaceae | sweet; plain | O/E | 1-58.5 | 20-43 |
| *Clematis montana* Buch.-Ham. ex DC./Clematidis caulis/川木通/CMU2021SWCmc | Plantae | Ranunculaceae | bitter; cold | O | 7 | - |
| *Ligusticum striatum* DC./Chuanxiong rhizoma/川芎/CMU2021SWCr | Plantae | Apiaceae | pungent; warm | O/E | 1.4-13.5 | 1-13.5 |
| *Salvia miltiorrhiza* Bunge/Salviae miltiorrhizae radix et rhizoma/丹參/CMU2021SWCmrr | Plantae | Lamiaceae | bitter; cold | O/E | 5.6 | 4 |
| *Actaea cimicifuga* L./Cimicifugae rhizoma/升麻/CMU2021SWAcir | Plantae | Ranunculaceae | pungent, sweet; cold | O/E | 1 | 15 |
| *Asparagus cochinchinensis* (Lour.) Merr./Asparagi radix/天門冬/CMU2021SWAcor | Plantae | Asparagaceae | sweet, bitter; cold | O/E | 6-37.5 | 18 |
| *Oroxylum indicum* (L.) Kurz/Oroxyli semen/木蝴蝶/CMU2021SWOis | Plantae | Bignoniaceae | bitter, sweet; cool | O | 1 | - |
| *Arctium lappa* L./Arctii fructus/牛蒡子/CMU2021SWAlf | Plantae | Compositae | bitter, sweet; cool | O | 6.8 | - |
| *Glehnia littoralis* F.Schmidt ex Miq./Glehniae radix/北沙參/CMU2021SWGlr | Plantae | Apiaceae | sweet, bitter; cold | O | 8.6-15 | - |
| *Polygonatum odoratum* (Mill.) Druce/Polygonati odorati rhizoma/玉竹/CMU2021SWPor | Plantae | Asparagaceae | sweet; cold | O/E | 5-18.8 | 10.5-11 |
| *Glycyrrhiza uralensis* Fisch./Glycyrrhiza radix et rhizoma/甘草/CMU2021SWGrr | Plantae | Leguminosae | sweet, plain | O/E | 0.4-23.2 | 6.5-12 |
| *Rehmannia glutinosa* (Gaertn.) DC. (prepared)/Rehmanniae radix praeparata/熟地黃/CMU2021SWRgrp | Plantae | Plantaginaceae | sweet; warm | O | 10-11.3 | - |
| *Bletilla striata* (Thunb.) Rchb.f./Bletillae rhizoma/白及/CMU2021SWBr | Plantae | Orchidaceae | bitter, sweet, astringent; cold | O/E | 1 | 1-37.5 |
| *Tremella fuciformis*/白木耳(銀耳) /CMU2021SWTf | Fungi | Tremellaceae | sweet, plain; plain | O | 6.5-12.2 | - |
| *Atractylodes macrocephala* Koidz./Atractylodis macrocephalae rhizoma/白朮/CMU2021SWAmr | Plantae | Compositae | bitter, sweet; warm | O/E | 1-17 | 1-22.5 |
| *Paeonia lactiflora* Pall. (white)/Paeoniae alba radix/白芍/CMU2021SWPra | Plantae | Paeoniaceae | bitter, sour; cold | O/E | 1-37.5 | 1-13 |
| *Ginkgo biloba* L./Ginkgo semen/白果/CMU2021SWGbs | Plantae | Ginkgoaceae | sweet, bitter, astringent; plain | E | - | 9 |
| *Angelica dahurica* (Hoffm.) Benth. & Hook.f. ex Franch. & Sav./Angelicae dahuricae radix/白芷/CMU2021SWAdr | Plantae | Apiaceae | pungent; warm | O/E | 1-37.5 | 1-74 |
| *Sauromatum giganteum* (Engl.) Cusimano & Hett/Typhonii rhizoma/白附子/CMU2021SWASgr | Plantae | Araceae | pungent; warm | O/E | 1.9 | 1-12 |
| *Lablab purpureus* (L.) Sweet/Lablab album semen/白扁豆/CMU2021SWLps | Plantae | Leguminosae | sweet; warm | O/E | 8.4-18.8 | 5 |
| *Bombyx mori* Linnaeus/Bymbyx batryticatus/白殭蠶/CMU2021SWBb | Animalia | Bombycidae | salty, pungent; plain | O/E | 7.5 | 4-19 |
| *Dictamnus albus* L./Dictamni cortex/白鮮皮/CMU2021SWDac | Plantae | Rutaceae | bitter; cold | E | - | 4-13 |
| *Ampelopsis japonica* (Thunb.) Makino/Ampelopsis radix/白蘞/CMU2021SWAr | Plantae | Vitaceae | bitter, pungent; cold | O/E | 1 | 1-37.5 |
| *Cinnamomum camphora* (L.) J. Presl/Borneolum/冰片/CMU2021SWCcb | Plantae | Dipterocarpaceae | pungent, bitter; cold | E | - | 2.5-11.25 |
| *Lilium lancifolium* Thunb./Lilii bulbus/百合/CMU2021SWLlb | Plantae | Liliaceae | sweet; cold | O | 11.6 | - |
| *Panax quinquefolius* L./Panacis quinquefolii radix/西洋參/CMU2021SWPqr | Plantae | Araliaceae | sweet, bitter; cool | O | 10.4 | - |
| *Paeonia* × *suffruticosa* Andrews/Moutan radicis cortex/牡丹皮/CMU2021SWPsc | Plantae | Paeoniaceae | bitter, pungent; cold | O/E | 7 | 3 |
| *Crassostrea gigas* (Thunberg)/Crassostreae concha/牡蠣/CMU2021SWCgc | Animalia | Ostreidae | salty, astringent; cold | O | 37.5 | - |
| *Gleditsia sinensis* Lam./Gleditsiae fructus/皂莢/CMU2021SWGsf | Plantae | Leguminosae | pungent, salty; warm | O/E | 1 | 6-23 |
| *Paeonia lactiflora* Pall. (red)/Paeoniae aubra radix/赤芍/CMU2021SWPlr | Plantae | Paeoniaceae | bitter; cold | O | 6.6-9.5 | - |
| *Saposhnikovia divaricata* (Turcz.) Schischk./Saposhnikoviae radix et rhizoma/防風/CMU2021SWCdrr | Plantae | Apiaceae | pungent, sweet; warm | O/E | 9.5 | 3 |
| *Tribulus terrestris* L./Tribuli Fructus/刺蒺藜/CMU2021SWTtf | Plantae | Zygophyllaceae | pungent, bitter; warm | O/E | 7.5 | 5-35 |
| *Notopterygium incisum* K.C.Ting ex H.T.Chang/ Notopterygii rhizoma et radix/羌活/CMU2021SWNir | Plantae | Apiaceae | pungent, bitter; warm | E | - | 1.6 |
| *Euryale ferox* Salisb./Euryales semen/芡實/CMU2021SWEfs | Plantae | Nymphaeaceae | sweet, astringent; plain | O | 1-24 | - |
| *Lonicera japonica* Thunb./Lonicerae japonicae flos/金銀花/CMU2021SWLjf | Plantae | Caprifoliaceae | sweet; cold | O | 11.3-13 | - |
| *Citrus* × *aurantium* L./Aurantii fructus Immaturus/枳實/CMU2021SWCaf | Plantae | Rutaceae | bitter, pungent, sour; cold | O | 8 | - |
| *Lycium chinense* Mill./Lycii fructus/枸杞子/CMU2021SWLf | Plantae | Solanaceae | sweet; plain | O/E | 1 | 17-24 |
| *Pteria martensii* (Dunker)/Margarita/珍珠/CMU2021SWPm | Animalia | Pteriidae | sweet, salty; cold | O/E | 1-5 | 1-37.5 |
| *Amomum villosum* Lour./Amomi fructus/砂仁/CMU2021SWAvf | Plantae | Zingiberaceae | pungent; warm | O/E | 1 | 7 |
| *Carthamus tinctorius* L./Carthami flos/紅花/CMU2021SWCtf | Plantae | Compositae | pungent; warm | E | - | 8 |
| *Ziziphus jujuba* Mill. (red)/Jujubae fructus (red)/紅棗/CMU2021SWJf | Plantae | Rhamnaceae | sweet; warm | O/E | 1-39.5 | 23-26 |
| *Bupleurum chinense* DC./Bupleuri radix/柴胡/CMU2021SWBcr | Plantae | Apiaceae | sweet; warm | O | 14 | - |
| *Trichosanthes kirilowii* Maxim./Trichosanthis radix/栝蔞根/CMU2021SWTkr | Plantae | Cucurbitaceae | pungent; bitter; cold | O/E | 11.6 | 8.2-36 |
| *Cinnamomum cassia* (L.) J.Presl (fructus)/ Cinnamomi fructus/桂子/CMU2021SWCcf | Plantae | Lauraceae | sweet; bitter; cold | O | 1 | - |
| *Cinnamomum cassia* (L.) J.Presl (ramulus)/Cinnamomi ramulus/桂枝/CMU2021SWCcr | Plantae | Lauraceae | pungent, sweet; warm | O/E | 2.9-9.5 | 4.5 |
| *Prunus persica* (L.) Batsch/Persicae semen/桃仁/CMU2021SWPps | Plantae | Rosaceae | bitter, sweet; plain | O/E | 12.5-24 | 7-12.5 |
| *Morus alba* L. (cortex)/Mori cortex/桑白皮/CMU2021SWMac | Plantae | Moraceae | sweet; cold | O/E | 7-7.7 | 8 |
| *Morus alba* L. (folium)/Mori folium/桑葉/CMU2021SWMaf | Plantae | Moraceae | sweet, bitter; cold | O/E | 9.4 | 12 |
| *Platycodon grandiflorus* (Jacq.) A.DC./Adenphorae radix/桔梗/CMU2021SWPgr | Plantae | Campanulaceae | bitter, pungent; plain | O/E | 5.2-14 | 18.5-20 |
| *Dioscorea collettii* var. *hypoglauca* (Palib.) S.J.Pei & C.T.Ting/Dioscoreae hypoglaucae rhizoma/萆薢/CMU2021SWDcr | Plantae | Dioscoreaceae | bitter; plain | O | 10-11 | - |
| *Wolfiporia extensa* (Peck) Ginns/Poria/白茯苓/CMU2021SWP | Plantae | Fungi | sweet, plain; plain | O/E | 1-.38.3 | 1-69 |
| *Wolfiporia extensa* (Peck) Ginns (poria cum pini radix)/Poria cum pini radix/茯神/CMU2021SWWe | Plantae | Fungi | sweet, plain; plain | O | 11.3 | - |
| *Nepeta tenuifolia* Benth./Nepetae herba/荊芥/CMU2021SWNth | Plantae | Lamiaceae | pungent; warm | O/E | 7 | 4 |
| *Gardenia jasminoides* J.Ellis/Gardeniae fructus/梔子/CMU2021SWGjf | Plantae | Rubiaceae | bitter; cold | O | 6.2-37.5 | - |
| *Lophatherum gracile* Brongn./Lophatheri herba/淡竹葉/CMU2021SWLgh | Plantae | Poaceae | sweet, plain; cold | O | 3.6 | - |
| *Ipomoea nil* (L.) Roth/Pharbitidis semen/牽牛子/CMU2021SWIns | Plantae | Convolvulaceae | bitter; cold | E | - | 6-11 |
| *Asarum heterotropoides* F.Schmidt f. *mandshuricum* (Maxim.) Kitag./Asari radix/細辛/CMU2021SWAhr | Plantae | Aristolochiaceae | pungent; warm | E | - | 1-5 |
| *Curcuma phaeocaulis* Valeton/Curcumae rhizoma/莪朮/CMU2021SWCpr | Plantae | Zingiberaceae | pungent, bitter; warm | O | 18.6 | - |
| *Forsythia suspensa* (Thunb.) Vahl/Forsythiae fructus/連翹/CMU2021SWFsf | Plantae | Oleaceae | bitter; cold | O | 5.4-11.3 | - |
| *Ophiopogon japonicus* (Thunb.) Ker Gawl./Ophiopogonis radix/麥門冬/CMU2021SWOr | Plantae | Asparagaceae | sweet, bitter; cold | O/E | 1-37.5 | 16-20 |
| *Broussonetia papyrifera* (L.) L'Hér. ex Vent./Broussonetiae fructus/楮實子/CMU2021SW Bpf | Plantae | Moraceae | sweet; cold | O/E | 1 | 12 |
| *Chrysanthemum morifolium* Ramat./Chrysanthemi flos/菊花/CMU2021SWCmf | Plantae | Compositae | sweet, bitter; cold | O/E | 3.8 | 4 |
| *Scutellaria baicalensis* Georgi/Scutellariae radix/黃芩/CMU2021SWSbr | Plantae | Lamiaceae | bitter; cold | O/E | 4.2-12.6 | 11 |
| *Phellodendron chinense* C.K.Schneid./Phellodendri cortex/黃蘗/CMU2021SWPcc | Plantae | Rutaceae | bitter; cold | O | 16.2 | - |
| *Astragalus propinquus* Schischkin/Astragali radix/黃耆/CMU2021SWApr | Plantae | Leguminosae | sweet; warm | O/E | 1-64 | 16-36 |
| *Coptis chinensis* Franch./Coptidis rhizoma/黃連/CMU2021SWCchr | Plantae | Ranunculaceae | bitter; cold | O | 5.2-7.5 | - |
| *Ziziphus jujuba* Mill. (black)/Jujubae fructus (black)/黑棗/CMU2021SWZjfb | Plantae | Rhamnaceae | sweet; warm | O | 10-72 | - |
| *Angelica sinensis* (Oliv.) Diels/Angelicae sinensis radix/當歸/CMU2021SWAsr | Plantae | Apiaceae | sweet, pungent; warm | O/E | 1-11 | 1-11 |
| *Pueraria montana* (Lour.) Merr./Puerariae radix/葛根/CMU2021SWPmr | Plantae | Leguminosae | sweet, pungent; cool | O/E | 29.8 | 5.8-22 |
| *Styphnolobium japonicum* (L.) Schott/Sophorae flos et flos Immaturus/槐花/CMU2021SWSjf | Plantae | Leguminosae | bitter; cold | O | 14.6 | - |
| *Vigna radiata* (L.) R.Wilczek/Mung bean/綠豆/CMU2021SWVrb | Plantae | Leguminosae | sweet; cold | O/E | 1-18.8 | 3.5-46 |
| *Nelumbo nucifera* Gaertn. (seman)/Nelumbinis semen/蓮子/CMU2021SWNns | Plantae | Nelumbonaceae | sweet, astringent; plain | O | 1-37 | - |
| *Nelumbo nucifera* Gaertn. (plumula)/Nelumbinis plumula/蓮子心/CMU2021SWNnp | Plantae | Nelumbonaceae | bitter; cold | E | - | 6 |
| *Nelumbo nucifera* Gaertn. (rhizoma)/Nelumbinis rhizoma/蓮藕/CMU2021SWNnr | Plantae | Nelumbonaceae | sweet, astringent; plain | O | 11.3 | - |
| *Alisma plantago-aquatica* subsp. orientale (Sam.) Sam./Alismatis rhizoma/澤瀉/CMU2021SWAprh | Plantae | Alismataceae | pungent, bitter; warm | O | 6.8 | - |
| *Angelica pubescens* Maxim./Angelicae pubescentis radix/獨活/CMU2021SWApra | Plantae | Apiaceae | pungent, bitter; warm | O/E | 2.5 | 4 |
| *Gentiana scabra* Bunge/Gentianae radix et rhizoma/龍膽草/CMU2021SWGsrr | Plantae | Gentianaceae | bitter; cold | O | 3.8-8.2 | - |
| *Santalum album* L./Santali albi lignum/檀香/CMU2021SWSal | Plantae | Santalaceae | pungent; warm | E | - | 1.4 |
| *Reynoutria multiflora* (Thunb.) Moldenke /Reynoutriae multiflorae radix/何首烏/CMU2021SWRmr | Plantae | Polygonaceae | bitter, sweet, astringent; warm | O | 13 | - |
| *Origanum vulgare* L./Menthae herba/薄荷/CMU2021SWOvh | Plantae | Lamiaceae | pungent; cool | O | 5 | - |
| *Coix lacryma-jobi* var. *ma-yuen* (Rom.Caill.) Stapf /Coicis semen/白薏仁/CMU2021SWCs | Plantae | Poaceae | sweet, plain; cool | O/E | 1-109 | 1-154 |
| *Fritillaria cirrhosa* D.Don/Fritillariae cirrhosae bulbus/川貝母/CMU2021SWFcb | Plantae | Liliaceae | bitter, sweet; cold | O | 3.6 | - |
| *Codonopsis pilosula* (Franch.) Nannf./Codonopsis radix黨參/CMU2021SWCpra | Plantae | Campanulaceae | sweet; plain | O | 7.6-11.3 | - |
| *Leonurus japonicus* Houtt./Leonuri herba/益母草/CMU2021SWLjh | Plantae | Lamiaceae | pungent, bitter; cold | E | - | 1 |
| *Prunus mume* (Siebold) Siebold & Zucc./Mume fructus/烏梅/CMU2021SWPmf | Plantae | Rosaceae | sour, astringent; plain | E | - | 1 |
| *Rosae rugosa* Thunb./Rosae rugosae flos玫瑰/CMU2021SWRrf | Plantae | Rosaceae | sweet, bitter; warm | O | 1-13 | - |
| O, oral; E, external. | | | | | | |

| **Table S3. 74 skin-whitening prescriptions composition and preparation.** | | | |
| --- | --- | --- | --- |
| **Formulation** | **Source** | **Species^1^, concentration.** | **Usage^2^** |
| TP-1-1 | Songshan Dist., Taipei City | Fvf, 4.5g. Cr, 13.5g. Por, 10.5g. Grr, 6.5g. Amr, 13g. Pra, 12.5g. Adr, 13g. Lf, 17g. Apr, 16g. Jf, 23g. Ccr, 4.5g. Pps, 12.5g. P, 17g. | O/E |
| TP-1-2 | Songshan Dist., Taipei City | Cr, 1g. Adr, 1g. Asr, 1g. Ljh, 1g. Pmf, 1g. | E |
| TP-1-3 | Songshan Dist., Taipei City | Amr, 1g. Adr, 1g. Sgr, 1g. Ahr, 1g. | E |
| TP-2 | Wanhua Dist., Taipei City | Adr, 19.1g. P, 38.3g. Cpr, 18.6g. Sbr, 10g. Pcc, 16.2g. | O |
| TP-3 | Neihu Dist., Taipei City | Pra, 15g. Adr, 11.25g. Sgr, 1.88g. Lps, 18.75g. Bb, 7.5g. Vrb, 18.75g. | O |
| TP-4 | Wanhua Dist., Taipei City | Grr, 4.9g. Amr, 8g. Pra, 6.8g. P, 6.2g. | O |
| TP-5 | Songshan Dist., Taipei City | Br, 4.5g. Adr, 4.5g. Ar, 7.5g. P, 13g. Cs, 1g. | E |
| TP-6 | Da’an Dist., Taipei City | Grr, 15.5g. Amr, 14.5g. Pra, 13.5g. P, 15g. | O |
| NTP-1 | Sanzhi Dist., New Taipei City | Amr, 5.8g. Adr, 7.6g. Tkr, 8.2g. Pmr, 5.8g. | E |
| NTP-2 | Zhonghe Dist., New Taipei City | Ar, 12g. Br, 11g. Amr, 12g. Pra, 11g. Adr, 11g. P, 11g. | E |
| NTP-3 | Sanchong Dist., New Taipei City | Glr, 15g. Grr, 17.5g. Amr, 17g. Apr, 19g. Pgr, 14g. Apra, 2.5g. | O |
| NTP-4-1 | Yonghe Dist., New Taipei City | Dr, 29.5g. Efs, 24g. P, 18g. Asr, 8g. Nns, 20.5g. Cs, 54g. | O |
| NTP-4-2 | Yonghe Dist., New Taipei City | Dr, 1g. Efs, 1g. Asr, 1g. Nns, 1g. Cs, 1g. | O |
| NTP-5 | Tamsui Dist., New Taipei City | Cr, 12g. Grr, 8g. Lf, 20g. Jf, 39.5g. Ccr, 9.5g. Apr, 14.5g. Zjfb, 72g. Asr, 10g. | O |
| NTP-6 | Sanzhi Dist., New Taipei City | Dr, 58.5g. Efs, 20g. P, 23.5g. Asr, 9g. Nns, 29g. Cs, 108.5g. | O |
| NTP-7 | Sanchong Dist., New Taipei City | Grr, 7g. Adr, 5g. Plr, 9.5g. Lf, 18.5g. Apr, 22.5g. Jf, 25.5g. Asr, 11g. | O |
| NTP-8 | Tucheng Dist., New Taipei City | Adr, 44g. Mac, 8g. Pgr, 18.5g. P, 33.5g. Pmr, 22g. | E |
| NTP-9 | Xizhi Dist., New Taipei City | Lf, 40g. Apr, 64g. Jf, 35.5g. Asr, 11g. | O |
| KL-1 | Xinyi Dist., Keelung City | Dr, 20g. Por, 11g. P, 21g. Or, 20g. Vrb, 3.5g. Cs, 35g. | E |
| TY-1-1 | Taoyuan Dist., Taoyuan City | Dr, 13.4g. Por, 6.6g. Tf, 12.2g. Or, 4.8g. Nns, 37g. | O |
| TY-1-2 | Taoyuan Dist., Taoyuan City | Lf, 1g. Jf, 1g. P, 1g. Or, 1g. Apr, 1g. Rrf, 1g. | O |
| TY-2-1 | Taoyuan Dist., Taoyuan City | Pgrr, 1.9g. Glr, 11.3g. Grr, 7.5g. Amr, 7.5g. Pra, 7.5g. Lps, 11.3g. Apr, 11.3g. Jf, 2g. P, 11.3g. Cmf, 3.8g. Cs, 26.3g. | O |
| TY-2-2 | Taoyuan Dist., Taoyuan City | Br, 1g. Amr, 1g. Pra, 1g. Adr, 1g. Ar, 1g. Pm, 1g. P, 1g. | O |
| TY-3 | Guishan Dist., Taoyuan City | Pra, 37.5g. Adr, 37.5g. Gjf, 37.5g. | O |
| TY-4 | Daxi Dist., Taoyuan City | Grr, 11g. Amr, 15g. Pra, 7g. Apr, 37g. Jf, 22.6g. Pps, 24g. P, 34.6g. Or, 22g. Cs, 56g. | O |
| HC-1 | East Dist., Hsinchu City | Adr, 6.8g. Sgr, 5.6g. P, 9.4g. Cs, 7.5g. | E |
| HC-2 | North Dist., Hsinchu City | Kgr, 7.4g. Cmc, 7g. Alf, 6.8g. Grr, 7g. Rgrp, 10.4g. Psc, 7g. Plr, 6.6g. Ljf, 13g. Pgr, 6.6g. Dcr, 11g. Gjf, 6.2g. Fsf, 5.4g. Sbr, 4.2g. Cchr, 5.2g. Aprh, 6.8g. Gsrr, 3.8g. | O |
| ML-1 | Toufen City, Miaoli County | Grr, 11g. Br, 13g. Adr, 11g. Ccb, 4g. Pps, 7g. Vrb, 46g. | E |
| TC-1-1 | North Dist., Taichung City | Br, 5g. Amr, 6g. Pra, 6g. Adr, 6g. Ar, 6g. Pm, 5g. P, 6g. | E |
| TC-1-2 | North Dist., Taichung City | Br, 5g. Amr, 5g. Adr, 6g. Bb, 4g. Ar, 5g. Ttf, 5g. P, 6g. | E |
| TC-1-3 | North Dist., Taichung City | Br, 5g. Amr, 7g. Adr, 6g. Sgr, 6g. Lps, 5g. Bb, 5g. Sdrr, 3g. Nir, 1.6g. Pm, 1.6g. P, 5g. Nth, 4g. Ins, 6g. Ahr, 5g. Vrb, 8g. Nnp, 6g. Apra, 4g. Sal, 1.4g. | E |
| TC-2-1 | Beitun Dist., Taichung City | Smrr, 4g. Br, 3g. Pra, 4g. Adr, 4g. Bb, 4g. Dac, 4g. Psc, 3g. P, 4g. Cmf, 4g. | E |
| TC-2-2 | Beitun Dist., Taichung City | Amr, 4g. Adr, 4g. gr, 3g. Ar, 4g. P, 3g. Ahr, 4g. Vrb, 4g. | E |
| TC-3-1 | Beitun Dist., Taichung City | Kgr, 7g. Acir, 15g. Br, 4g. Adr, 4g. Gsf, 23g. Avf, 7g. P, 4g. Bpf, 12g. | E |
| TC-3-2 | Beitun Dist., Taichung City | Br, 10g. Adr, 11g. Sgr, 4g. Bb, 9g. Gsf, 6g. Ttf, 9g. P, 4g. Ins, 10g. | E |
| TC-4 | North Dist., Taichung City | Ar, 13g. Br, 10g. Amr, 13g. Pra, 13g. Adr, 14g. Bb, 14g. P, 12g. | E |
| TC-5 | North Dist., Taichung City | Amr, 13g. Adr, 11g. Tkr, 13g. P, 12g. Sbr, 11g. | E |
| CH-1 | Hemei Township, Changhua County | Amr, 14g. Pra, 12g. Adr, 13g. Bb, 13g. Dac, 13g. Pm, 16g. P, 20g. | E |
| CH-2 | Hemei Township, Changhua County | Acor, 18g. Grr, 7g. Br, 19g. Adr, 9g. P, 23g. | E |
| CH-3 | Hemei Township, Changhua County | P, 13g. Cs, 154g. | E |
| CH-4 | Changhua City, Changhua County | Amr, 16g. Pra, 18g. Adr, 11g. Lf, 16g.Apr, 18g.Dcr, 10g.P, 22g. | O |
| CH-5 | Changhua City, Changhua County | Pgrr, 12g.Lf, 24g.Apr, 36g.Jf, 26g.Or, 16g. | E |
| NT-1-1 | Puli Township, Nantou County | Pgrr, 8g. Cr, 9g. Grr, 12g. Amr, 9g. Gbs, 9g. Adr, 9g. Maf, 12g. P, 10g. Asr, 9g. | E |
| NT-1-2 | Puli Township, Nantou County | Saf, 12g. Br, 12g. Adr, 12g. Sgr, 12g. Bb, 12g. Ttf, 12g. Gsf, 14g. P, 12g. Ins, 11g. Vrb, 11g. | E |
| NT-2 | Puli Township, Nantou County | Pgrr, 11g. Grr, 11g. Ctf, 8g. Asr, 11g. Pmr, 11g. Cs, 11g. | E |
| YLC-1 | Huwei Township, Yunlin County | Br, 4g. Amr, 9g. Pra, 7g. Adr, 10g. Bb, 4g. Ar, 6g. P, 7g. | E |
| YLC-2 | Sihu Township, Yunlin County | Br, 9g. Adr, 23g. Bb, 19g. Ar, 19g. Pm, 8g. Pgr, 20g. P, 22g. | E |
| YLC-3 | Gukeng Township, Yunlin County | Glr, 8.6g. Por, 7.2g. Grr, 0.4g. Lps, 8.4g. Tkr, 11.6g. Maf, 9.4g. Or, 10.8g. Cs, 18.8g. | O |
| CY-1-1 | East Dist., Chiayi City | Pgrr, 11.5g. Dr, 35.5g. Cr, 1.4g. Ois, 1g.Lf, 17g. Ar, 25.5g. Jf, 7g. Ccf, 1g. Ccr, 2.9g. Zjfb, 10g. | O |
| CY-1-2 | East Dist., Chiayi City | Br, 11g. Amr, 11.5g. Adr, 11g. Ar, 11.5g. Ttf, 11g. | E |
| TN-1 | South Dist., Tainan City | Cr, 8.3g. Por, 7.9g. Grr, 7.1g. Rgrp, 11.3g. Amr, 6.4g. Adr, 9g. Apr, 12.4g. Jf, 18.8g. P, 13.1g. Asr, 9.4g. | O |
| TN-2-1 | Xinshi Dist., Tainan City | Cr, 13.4g. Grr, 23.2g. Amr, 11.6g. Pra, 20g. Adr, 34.6g. P, 24g. Gjf, 10.8g. Fsf, 7.4g. Sbr, 12.6g. Asr, 11g. Pmr, 29.8g. Sjf, 14.6g. | O |
| TN-2-2 | Xinshi Dist., Tainan City | Por, 18.8g. Grr, 7.5g. Pra, 18.8g. Cgc, 37.5g. Bcr, 14g. We, 11.3g. | O |
| TN-3 | Guiren Dist., Tainan City | Grr, 3.8g. Pra, 11.3g. Adr, 7.5g. Ttf, 7.5g. Ljf, 11.3g. Fsf, 11.3g. Or, 18.8g. | O |
| TN-4 | Jiali Dist., Tainan City | Glr, 11.1g. Grr, 5.4g. Amr, 8g. Pra, 10.2g Llb, 11.6g. Mac, 7.7g. Pgr, 5.2g. P, 8g. Or, 7.4g. Asr, 8g. Gsrr, 8.2g. Fcb, 3.6g. Cpra, 8g. | O |
| TN-5 | West Central Dist., Tainan City | Dr, 15.2g. Cr, 8.4g. Rgrp, 10g. Pra, 10.2g Efs, 18.8g. Apr, 9g. Jf, 12.2g. P, 15.8g. Asr, 10.4g. Nns, 13.2g. Cs, 25g. Cpra, 7.6g. | O |
| TN-6 | East Dist., Tainan City | Dr, 36.5g. Adr, 36.5g. Tkr, 36g. P, 36.5g. | E |
| KH-1 | Qianjin Dist., Kaohsiung City | Br, 37.5g. Adr, 37.5g. Ar, 37.5g. P, 37.5g. Cs, 37.5g. | E |
| KH-2 | Lingya Dist., Kaohsiung City | Dr, 6g. Acor, 6g. Por, 5g. Tf. 6.5g. Adr, 5.5g. Pm, 5g. Mac, 7g. P, 8g. Or, 6g. Cs, 7g. | O |
| KH-3-1 | Lingya Dist., Kaohsiung City | Dr, 22g. Efs, 21g. Lf, 10.5g. P, 14g. Asr, 7g. Nns, 21.5g. Cs, 30g. | O |
| KH-3-2 | Lingya Dist., Kaohsiung City | Kgr, 1g. Acir, 1g. Br, 1g. Adr, 1g. Gsf, 1g. Avf, 1g. Bpf, 1g. Vrb, 1g. | O |
| KH-4 | Sanmin Dist., Kaohsiung City | Acor, 11.3g. Or, 11.3g. Nnr, 11.3g. | O |
| KH-5-1 | Zuoying Dist., Kaohsiung City | Br, 2.2g. Amr, 11.8g. Pra, 10g. Adr, 8.4g. P, 18g. | E |
| KH-5-2 | Zuoying Dist., Kaohsiung City | Br, 1g. Amr, 1g. Pra, 1g. Adr, 1g. Ar, 1g. Pm, 1g. P, 1g. | E |
| KH-6 | Qianjin Dist., Kaohsiung City | Ar, 2.5g. Br, 4.5g. Amr, 3g. Pra, 4g. Adr, 3.5g. P, 5.5g. | E |
| KH-7 | Zuoying Dist., Kaohsiung City | Ar, 11.3g. Br, 11.3g. Amr, 11.3g. Pra, 11.3g. Adr, 11.3g. Pm, 11.3g. P, 11.3g. | E |
| KH-8 | Xiaogang Dist., Kaohsiung City | Smrr, 5.6g. Pqr, 10.4g. P, 6.2g. Lgh, 3.6g. Or, 8.6g. | O |
| KH-9 | Renwu Dist., Kaohsiung City | Acor, 20g. Rrf, 13g. Jf, 39.5g. Rmr, 13g. Cs, 21.5g. | O |
| PT-1 | Gaoshu Township, Pingtung County | Dr, 43g. Amr, 22.5g. Adr, 74g. Bb, 12.5g. Ttf, 35g. P, 69g. Gsrr, 22.5g. | E |
| YL-1 | Yilan City, Yilan County | Br, 2.5g. Amr, 6.5g. Pra, 2.5g. Adr, 4g. Ar, 2g. P, 6.5g. | E |
| YL-2 | Yilan City, Yilan County | Dr, 18.8g. Acor, 37.5g. Adr, 7.5g. Lf, 18.8g. Apr, 11.3g. P, 37.5g. Or, 37.5g. Asr, 7g. Cs, 37.5g. Cpra, 11.3g. | O |
| HL-1 | Hualien City, Hualien County | Cr, 8g. Grr, 6.5g. Adr, 6.5g. Sdrr, 9.5g. Caf, 8g. Pgr, 9g. Nth, 7g. Gjf, 7.5g. Fsf, 7g. Cchr, 7.5g. Sbr, 9g. Ovh, 5g. Cs, 23g. | O |
| HL-2-1 | Ji’an Township, Hualien County | Br, 37.5g. Adr, 37.5g. Pm, 37.5g. P, 37.5g. | E |
| HL-2-2 | Ji’an Township, Hualien County | Lf, 1g. | O |
| ^1^Acir, *Actaea cimicifuga* L./Cimicifugae rhizome; Acor, *Asparagus cochinchinensis* (Lour.) Merr.; Adr, *Angelica dahurica* (Hoffm.) Benth. & Hook.f. ex Franch. & Sav.; Ahr, *Asarum heterotropoides* F.Schmidt f. *mandshuricum* (Maxim.) Kitag.; Alf, *Arctium lappa* L.; Apr, *Astragalus propinquus* Schischkin; Apra, *Angelica pubescens* Maxim.; Aprh, *Alisma plantago-aquatica* subsp. orientale (Sam.) Sam.; Ar, *Ampelopsis japonica* (Thunb.) Makino; Asr, *Angelica sinensis* (Oliv.) Diels; Avf, *Amomum villosum* Lour.; Bb, *Bombyx mori* Linnaeus; Bcr, *Bupleurum chinense* DC.; Bpf, *Broussonetia papyrifera* (L.) L'Hér. ex Vent.; Br, *Bletilla striata* (Thunb.) Rchb.f.; Caf, *Citrus* × *aurantium* L.; Ccb, *Cinnamomum camphora* (L.) J. Presl; Cchr, *Coptis chinensis* Franch.; Ccf, *Cinnamomum cassia* (L.) J.Presl (fructus); Ccr, *Cinnamomum cassia* (L.) J.Presl (ramulus); Cdrr, *Saposhnikovia divaricata* (Turcz.) Schischk.; Cgc, *Crassostrea gigas* (Thunberg); Cmc, *Clematis montana* Buch.-Ham. ex DC.; Cmf, *Chrysanthemum morifolium* Ramat.; Cmrr, *Salvia miltiorrhiza* Bunge; Cpr, *Curcuma phaeocaulis* Valeton; Cpra, *Codonopsis pilosula* (Franch.) Nannf; Cr, *Ligusticum striatum* DC.; Cs, *Coix lacryma-jobi* var. *ma-yuen* (Rom.Caill.) Stapf; Ctf, *Carthamus tinctorius* L.; Dac, *Dictamnus albus* L.; Dcr, *Dioscorea collettii* var. *hypoglauca* (Palib.) S.J.Pei & C.T.Ting; Dr, *Dioscorea polystachya* Turcz.; Efs, *Euryale ferox* Salisb.; Fcb, *Fritillaria cirrhosa* D.Don/Fritillariae cirrhosae bulbus; Fsf, *Forsythia suspensa* (Thunb.) Vahl; Fvf, *Foeniculum vulgare* Mill.; Gbs, *Ginkgo biloba* L.; Gjf, *Gardenia jasminoides* J.Ellis; Glr, *Glehnia littoralis* F.Schmidt ex Miq.; Grr, *Glycyrrhiza uralensis* Fisch.; Gsf, *Gleditsia sinensis* Lam.; Gsrr, *Gentiana scabra* Bunge; Ins, *Ipomoea nil* (L.) Roth; Jf, *Ziziphus jujuba* Mill. (red); Kgr, *Kaempferia galanga* L.; Lf, *Lycium chinense* Mill.; Lgh, *Lophatherum gracile* Brongn.; Ljf, *Lonicera japonica* Thunb.; Ljh, *Leonurus japonicus* Houtt.; Llb, *Lilium lancifolium* Thunb.; Lps, *Lablab purpureus* (L.) Sweet; Mac, *Morus alba* L. (cortex); Maf, *Morus alba* L. (folium); Nir, *Notopterygium incisum* K.C.Ting ex H.T.Chang; Nnp, *Nelumbo nucifera* Gaertn. (plumula); Nnr, *Nelumbo nucifera* Gaertn. (rhizoma); Nns, *Nelumbo nucifera* Gaertn. (seman); Nth, *Nepeta tenuifolia* Benth.; Ois, *Oroxylum indicum* (L.) Kurz; Or, *Ophiopogon japonicus* (Thunb.) Ker Gawl.; Ovh, *Origanum vulgare* L.; P, *Wolfiporia extensa* (Peck) Ginns; Pcc, *Phellodendron chinense* C.K.Schneid.; Pgr, *Platycodon grandiflorus* (Jacq.) A.DC.; Pgrr, *Panax ginseng* C.A.Mey.; Plr, *Paeonia lactiflora* Pall. (red); Pm, *Pteria martensii* (Dunker); Pmf, *Prunus mume* (Siebold) Siebold & Zucc.; Pmr, *Pueraria montana* (Lour.) Merr.; Por, *Polygonatum odoratum* (Mill.) Druce; Pps, *Prunus persica* (L.) Batsch; Pqr, *Panax quinquefolius* L.; Pra, *Paeonia lactiflora* Pall. (white); Psc, *Paeonia* × *suffruticosa* Andrews; Rgrp, *Rehmannia glutinosa* (Gaertn.) DC. (prepared); Rmr, *Reynoutria multiflora* (Thunb.) Moldenke; Rrf, *Rosae rugosa* Thunb.; Saf, *Syzygium aromaticum* (L.) Merr. & L.M.Perry; Sal, *Santalum album* L.; Sbr, *Scutellaria baicalensis* Georgi; Sgr, *Sauromatum giganteum* (Engl.) Cusimano & Hett; Sjf, *Styphnolobium japonicum* (L.) Schott; Tf, *Tremella fuciformis;* Tkr, *Trichosanthes kirilowii* Maxim.; Ttf, *Tribulus terrestris* L.; Vrb, *Vigna radiata* (L.) R.Wilczek; We, *Wolfiporia extensa* (Peck) Ginns (poria cum pini radix); Zjfb, *Ziziphus jujuba* Mill. (black).  ^2^O, oral; E, external. | | | |
